# ArfGAP3 Protects Mitochondrial Function and Promotes Autophagy Through Rab5a‐Mediated Signals in Ageing Skeletal Muscle

**DOI:** 10.1002/jcsm.13725

**Published:** 2025-02-17

**Authors:** Mao Chen, Xiaoyu Huang, Bingshu Li, Ya Xiao, Liying Chen, Fangyi Zhu, Shasha Hong, Jianming Tang, Suting Li, Jie Min, Wenyi Jin, Yubiao Zhang, Lian Yang, Yang Li, Shufei Zhang, Li Hong

**Affiliations:** ^1^ Department of Gynecology and Obstetrics Renmin Hospital of Wuhan University Wuhan Hubei China; ^2^ Hubei Provincial Clinical Medical Research Center for Pelvic Floor Disease Wuhan Hubei China; ^3^ Department of Biomedical Sciences City University of Hong Kong Hong Kong Special Administrative Region China; ^4^ Department of Orthopedics Renmin Hospital of Wuhan University Wuhan Hubei China

**Keywords:** ageing, ArfGAP3, autophagy, pelvic floor muscle, Rab5a

## Abstract

**Background:**

Few researches have investigated the molecular mechanism responsible for the age‐related loss of the pelvic floor muscle (PFM) mass and functionality—a pivotal contributor to pelvic organ prolapse and diminished physical well‐being. ADP ribosylation factor GTPase activating protein 3 (ArfGAP3) is a member of ArfGAPs, which regulates the vesicular trafficking pathway and intracellular proteins transporting. However, its effects on skeletal muscle ageing remain largely unknown.

**Methods:**

Mouse models of natural ageing and D‐gal (D‐galactose)–induced ageing were subject to analyse the structure, function and pathological alterations of the PFM and the expression of ArfGAP3. Stable ArfGAP3 knockdown and overexpression C2C12 cell lines were established to investigate the anti‐senescence effects of ArfGAP3 and the underlying mechanisms in ageing process, complemented by Rab5a genetic intervention and mRFP‐GFP‐LC3 adenoviral particles transfection. In vivo experiments entailed ArfGAP3 overexpression in mice alongside autophagy inhibitor treatment, with assessments encompassing tissue mass, bladder leak point pressure (BLPP), submicroscopic structure, antioxidative stress system and muscle regeneration.

**Results:**

Aged (24‐month‐old) mice exhibited significant physiological alterations in PFMs, including decreased muscle mass, diminished cross‐sectional area (CSA), deteriorated supporting function (as evidenced by reduced BLPP), impaired autophagy and increased levels of oxidative stress (*p* < 0.001). Utilizing ageing C2C12 model, we observed a dose‐dependent relationship between D‐gal induction and cellular senescence, impaired differentiation and mitochondrial damage. Remarkably, the expression levels of ArfGAP3 were markedly downregulated in both in vitro and in vivo ageing models. Knockdown of ArfGAP3 exacerbated impaired differentiation potential and induced aberrant mitochondrial morphology and functional dysfunction in ageing C2C12 myoblasts, whereas ArfGAP3 overexpression largely mitigated these effects. Mechanistically, our findings revealed an interplay between ArfGAP3 and Rab5a, indicating their coordinated regulation. ArfGAP3‐mediated activation of Rab5a‐associated autophagy and IRS1‐AKT‐mTOR signalling pathways during cellular senescence and myogenesis was identified, leading to enhanced autophagic flux and improved resistance to oxidative stress. In vivo, ArfGAP3 overexpression ameliorated D‐gal–induced loss of muscle mass and function, while promoting antioxidant responses and muscle regeneration in mice. However, these protective effects of ArfGAP3 overexpression were extinguished by autophagy inhibition.

**Conclusions:**

Our study uncovers the significant role of ArfGAP3 in enhancing differentiation capacity and mitochondrial function through mediating Rab5a expression to activate IRS1‐AKT‐mTOR signalling pathways and promote autophagy during the ageing process. These findings underscore the potential of ArfGAP3 as a promising therapeutic target for ameliorating the decline in skeletal muscle function associated with ageing.

## 1 Introduction

The rapid ageing of populations worldwide has escalated the global burden of late‐life disease associated with various types of physiological deterioration [1]. Age‐related degenerative loss of skeletal muscle mass and function has been the leading cause of frailty‐to‐disability transition in the aged population [2]. In particular, pelvic floor disorders (PFDs) pose significant health challenges for older women, including urinary incontinence, faecal incontinence and pelvic organ prolapse, which have profound negative impacts on quality of life and economic costs. These disorders are often attributed to the weakening of connective tissues, pelvic floor muscle (PFM), or both [3, 4]. Age‐related dysfunction of PFM has been identified as one of the major inciting and promoting events for PFDs, which are associated with degenerative alterations involving a decrease in the muscle stem cell (MuSC) pool, intramuscular lipid accumulation and decreased physiologic cross‐sectional area after 60 years of age [5, 6, 7]. However, the underlying pathophysiology of PFDs and the exact cause of age‐related PFM dysfunction remain complex and elusive.

Ageing is a complex biological process characterized by the accumulation of hallmark features, including mitochondrial dysfunction, cellular senescence, imbalanced metabolism, stem cell exhaustion, decreased autophagy function and immune ageing. A growing body of evidence has placed mitochondria at the centre of skeletal muscle ageing, while basal autophagy is essential for maintaining the quiescent state of stem cells (Supporting Information S6, Supporting Information S3, Supporting Information S9). It has been well documented that increased oxidative stress mediated by the accumulation of reactive oxygen species (ROS) damage over time causes mitochondrial dysfunction, which leads to age‐related skeletal muscle abnormalities [8, 9]. Autophagy is an evolutionarily conserved, lysosome‐dependent catabolic process that removes damaged organelles, misfolded proteins and lipid droplets, playing an essential role in maintaining cellular homeostasis. However, the efficiency of autophagy declines with age, which results in further aggravation of intracellular derangements [10].

ADP ribosylation factor GTPase activating protein 3 (ArfGAP3) is a GTPase‐activating protein that associates with the Golgi apparatus and regulates the vesicular trafficking pathway [11]. The Golgi complex is recognized as an important site of key autophagy regulators [12], and functional impairments in vesicular trafficking pathways underlie the pathogenesis of many human diseases [13]. Important insights have been gained regarding ArfGAP3 regulation of the transport of cation‐independent mannose 6‐phosphate (M6P) receptor in the post‐Golgi compartment [14], which functions to recognize M6P‐tagged proteins being delivered from the trans‐Golgi network to the endolysosomal system [15]. ArfGAP3 colocalizes the most with Rab5 [14], a marker for early endosome involvement in vesicle generation through fusion and fission and autophagosome formation [16]. Interestingly, ArfGAP3 has been reported to significantly predict the clinical outcome of prostate cancer (PC) patients [17] and is involved in promoting PC cell proliferation and migration as a novel androgen‐regulated gene [18]. Androgen deprivation therapy (ADT) for PC selectively decreased the volume of the levator ani muscle (LAM) [19], whereas testosterone administration has been shown to increase LAM hypertrophy and improve stress incontinence in a rodent model [20]. We previously reported that ArfGAP3 was significantly upregulated in the repair process after skeletal muscle injury in mice by analysing several Gene Expression Omnibus datasets and damage repair of LAM induced by mechanical trauma [21]. We also observed that ArfGAP3 significantly increased on days 1, 3 and 5 of differentiation culture and peaked on day 3 [21]. In this study, we speculated that the expression of ArfGAP3 was correlated with changes in PFM mass and are the first to report the important role of ArfGAP3 in the ageing process.

## 2 Materials and Methods

### 2.1 Animals

All virgin female C57BL/6J mice were provided by the animal experimental centre of Renmin Hospital of Wuhan University. All of the procedures involving animals were approved by the Institutional Animal Care and Use Committee of Renmin Hospital of Wuhan University. To establish an aged mouse model, 10‐week‐old mice were randomly divided into four groups (*n* = 15) and raised until they were 3, 8, 12 and 24 months old. To establish a D‐gal–induced ageing mouse model, 3‐month‐old mice were randomly divided into three groups depending on treatment time, namely, the normal control (Con) without treatment, D‐gal administration for 2 months (D‐gal‐2mon) and D‐gal administration for 4 months (D‐gal‐4mon) groups, with 15 mice in each group. D‐gal administration was performed with intraperitoneal injection of D‐gal at a daily dose of 150 mg/kg body weight dissolved in 0.9% saline. Mice from the Con group were administered saline at an identical volume. To achieve ArfGAP3 overexpression in the D‐gal–induced model experiments for 4 months (or 16 weeks), empty vector lentivirus vehicle or ArfGAP3‐overexpressing lentivirus (1 × 108 IU/mL) was injected into the tail vein of the mice (10 μL) twice a week for 4 weeks starting at week 12. For 3‐MA studies, mice in different groups were intraperitoneally injected with 3‐MA (30 mg/kg). D‐gal treatment protocols were performed as described above. D‐gal was ordered from Source Leaf Biotech (Shanghai, China). All lentiviruses were obtained from Shanghai GeneChem Co., Ltd. The body weights of the mice were monitored on the first week and the day after the final treatment. Urodynamic tests were conducted after mice were anaesthetised to measure the maximum cystometric capacity (MCC) and BLPP with the PowerLab data acquisition and analysis system (ad Instruments, Australia) as described previously (Supporting Information S7). Then the muscle mass was isolated for weighing and further analysis.

### 2.2 Cell Treatment and Transfection

To induce cell senescence, C2C12 myoblasts were treated with different concentrations (0, 20 and 40 g/L) of D‐gal for 48 h. To gain insight into the effects of ArfGAP3 and Rab5a on C2C12 myoblasts, lentiviral vectors were designed and generated by Shanghai GeneChem Co., Ltd. for knockdown or overexpression of ArfGAP3 and overexpression of Rab5a. For ArfGAP3 knockdown, the RNAi lentiviral vector targeting the ArfGAP3 sequence (sh‐ArfGAP3) and the negative control (sh‐NC) insertion sequence were transfected into myoblasts using Lipofectamine 2000 (Invitrogen, USA) at an MOI ratio of 50 according to the manufacturer's protocol. The recombinant plasmid of lentiviral vectors for ArfGAP3 or Rab5a overexpression (OE‐ArfGAP3 or OE‐Rab5a) and the empty vector as a negative control (NC) were transfected into C2C12 myoblasts. C2C12 myoblasts were cultured in PM and lentiviral supernatant (two rounds separated 16 h, MOI = 20) at 37°C. For Rab5a knockdown, C2C12 cells in different groups at 30%–40% confluency were transfected with 100 nM of siRNA (Shanghai GeneChem Co.) using Lipofectamine RNAiMAX (Life Technologies) according to the manufacturer's protocol.

### 2.3 Statistical Analyses

All data are presented as the mean ± SD from at least three independent experiments and were analysed with GraphPad Prism 8.0 software. We performed two‐tailed Student's t test and one‐way ANOVA for statistical analyses with two groups and more than two groups, respectively. Pearson's correlation coefficient was used to evaluate the correlation between Rab5a and ArfGAP3 expression. A *p* value < 0.05 was considered statistically significant.

## 3 Results

### 3.1 ArfGAP3 Was Downregulated With Skeletal Muscle Ageing

**FIGURE 1:**
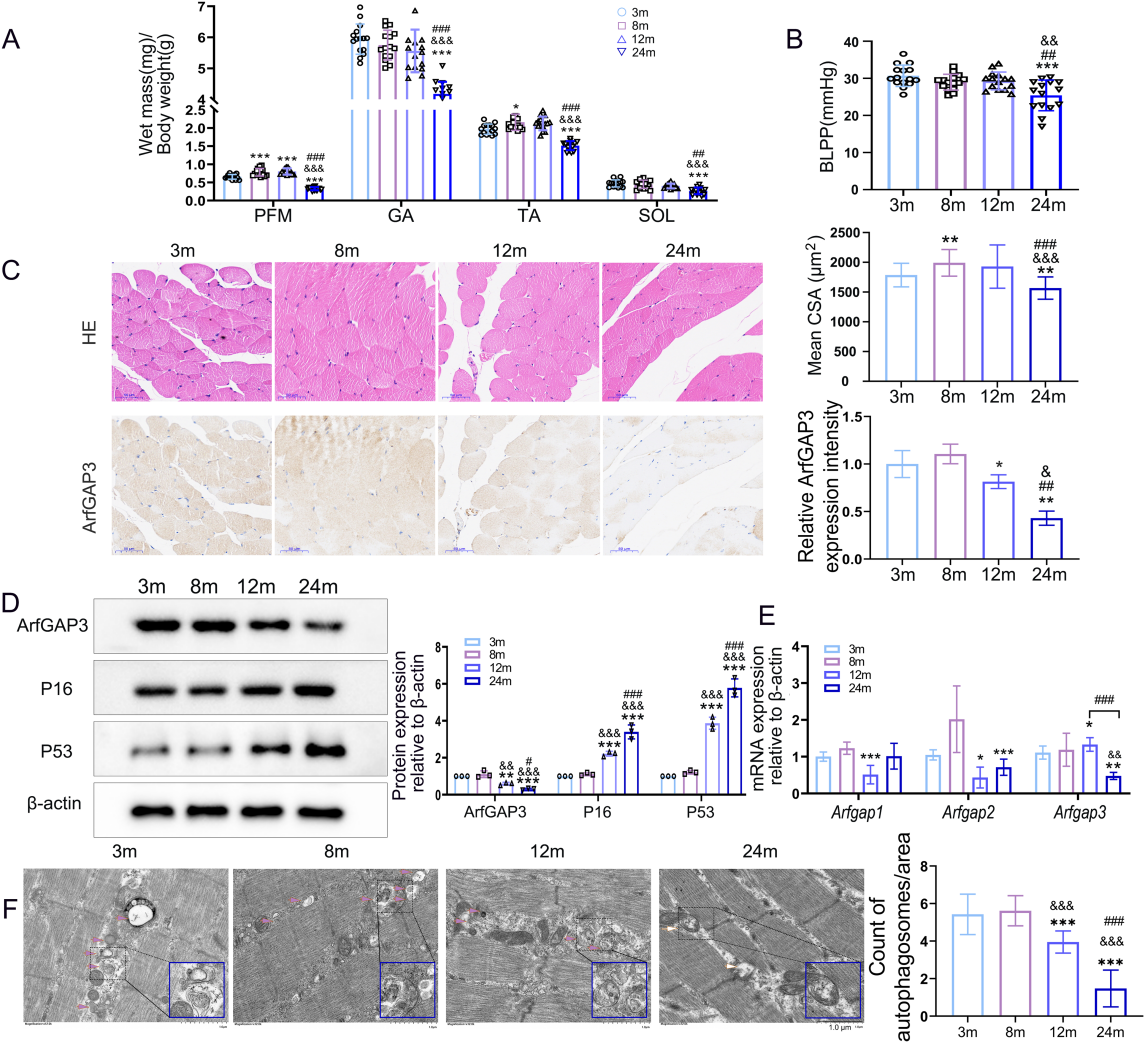
ArfGAP3 was downregulated with skeletal muscle ageing. (A) Weights of four types of muscles (PFM: pelvic floor muscle, GA: gastrocnemius, TA: tibialis anterior and SOL: soleus) in mice from four different groups (*n* = 15). (B) The average bladder leak point pressure (BLPP) values from the mice in the four groups (*n* = 15). (C) H&E staining of PFM myofibres (magnification 200×; scale bar = 50 μm) alongside measurement of the mean cross‐sectional area (CSA) of PFM fibres via ImageJ software (upper panels) is presented. The analysis included seven mice, with CSA measurement of more than 50 muscle fibres per mouse as evaluated through HE staining (*n* = 7). The lower panel shows IHC images of ArfGAP3 in PFM of mice (magnification 200×; scale bar = 50 μm) and the quantitative assessment of ArfGAP3 expression intensity using ImageJ software. (D) Western blot analysis and quantification of ArfGAP3, P16 and P53 protein levels in mouse PFM. (E) qRT‐PCR analysis of the expression of *Arfgap1*, *Arfgap2* and *Arfgap3*. *β‐actin* was used as the loading control. (F) Representative images of the ultrastructure showing muscle bundles, autophagosomes (pink arrows) and abnormal mitochondrial organelles (white arrows) of PFM from the four groups (magnification = × 12.0k). Quantification of the mean number of autophagosomes per area. All data were presented as mean ± SD and analysed using one‐way ANOVA. *p < 0.05/**p < 0.01/***p < 0.001 versus 3 months group; ^&^ *p* < 0.05/^&&^ *p* < 0.01 /^&&&^ *p* < 0.001 versus 8 months group; ^#^ *p* < 0.05/^##^ *p* < 0.01/^###^ *p* < 0.05 versus 12 months group.

Our previous findings suggested that ArfGAP3 might facilitate the repair process in PFM affected by mechanical trauma [21]. Given the substantial influence of ageing on PFM deterioration and the development of PFDs, we examined the expression of ArfGAP3 in human skeletal muscle ageing utilizing the GSE117525 dataset (Supporting Information: Materials and Methods). Our analysis revealed a decreased expression of ArfGAP3 in the skeletal muscle of frail elderly individuals (Figure S1A). To investigate the alterations in PFM, we raised mice to 24 months of age to establish a natural ageing model. The body weight of mice decreased significantly around 24 months of age (Figure S1B). We then analysed the muscle mass of fast [tibialis anterior (TA) muscle], mixed‐fibre‐type [gastrocnemius (GA) and PFM] and slow (soleus, SOL) muscle. Consistent with body weight changes, the skeletal muscle mass of aged mice was also significantly decreased (Figure 1A). Given that PFM dysfunction may contribute to clinical manifestations such as stress urinary incontinence, typically characterized by a reduced BLPP, we also examined the MCC and BLPP of mice to assess the supporting function of PFM. The results indicated that there were no apparent changes in MCC (Figure S1C), however, BLPP significantly decreased in 24‐month‐old mice (Figure 1B). These results can be explained by the age correspondence between humans and mice, namely, that 24‐month‐old mice are equivalent to over 65‐year‐old humans [22], a stage at which the incidence of PFDs increases rapidly. Moreover, the cross‐sectional area (CSA) of PFM was also decreased at 24 months (Figure 1C, top panels). The IHC images showed that ArfGAP3 expression was significantly decreased at the age of 12 and 24 months (Figure 1C, bottom panels). Additionally, the relative protein levels of the ageing‐related markers p53 and p16 were significantly increased, while ArfGAP3 was downregulated at the age of 12 and 24 months (Figure 1D). The mRNA levels of Arfgap1, Arfgap2 and Arfgap3 showed a specific decrease in Arfgap3 (Figure 1E) and aggravated oxidative stress damage in ageing PFM, characterized by increased expression of Nox2 and Nox4 (Figure S1D). To comprehensively evaluate the antioxidant response of PFM, we demonstrated a significant increase in malondialdehyde (MDA) levels within aged PFM, concomitant with a notable reduction in the activities of superoxide dismutase (SOD), glutathione S‐transferase (GST) and catalase (CAT) (Figure S1E). Moreover, we observed a complete absence of autophagosomes and the presence of large, misshapen mitochondria with disorganized cristae in PFM from 24‐month‐old mice (Figure 1F and Figure S1F). The arrangement of muscle bundles appeared distorted and visibly less organized in ageing PFM compared with the samples from younger mice (Figure 1F). These results suggest that ageing contributed to more pronounced degeneration in PFM along with decreased ArfGAP3 expression.

### 3.2 ArfGAP3 Was Downregulated in Aged Skeletal Muscle and Senescent C2C12 Myoblasts Induced by D‐Gal

**FIGURE 2:**
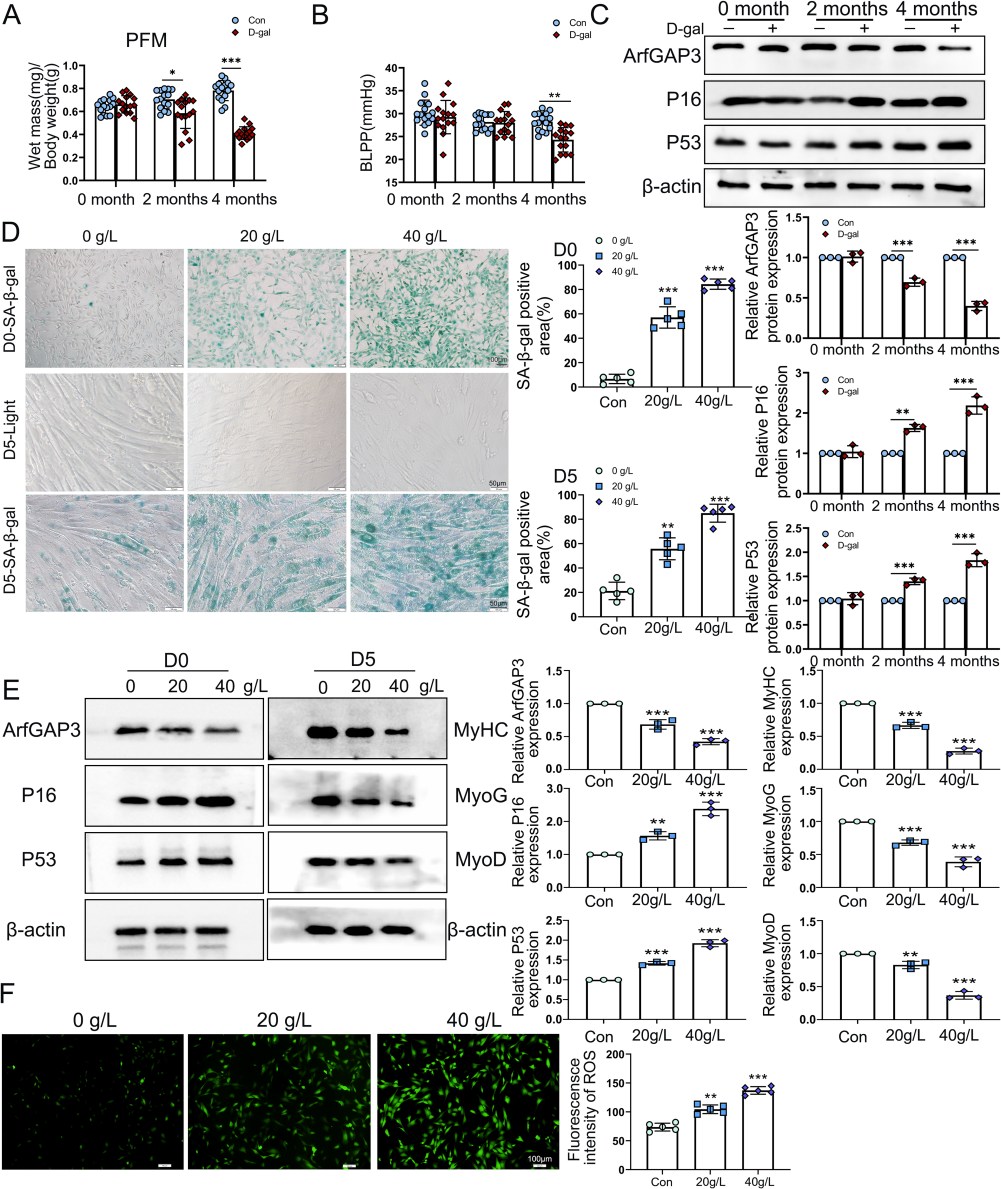
ArfGAP3 was downregulated in aged skeletal muscle and senescent C2C12 myoblasts induced by D‐gal. (A) Wet weights of PFM muscle samples (*n* = 15). (B) Average bladder leak point pressure (BLPP) values of mice (*n* = 15). (C) Western blots and quantification of ArfGAP3, P16 and P53 protein levels in mouse PFM. (D) SA‐β‐gal staining and quantification for C2C12 myoblasts treated with different concentrations of D‐gal on day 0 (D0) (top panels) and day 5 (D5) (bottom panels) after differentiation induction, and light microscopic images of unstained C2C12 myotubes. (E) Western blot analysis and quantification of ArfGAP3, P16 and P53 proteins in C2C12 myoblasts after D‐gal treatment for 48 h (D0) and protein levels of MyHC, MyoG and MyoD after differentiation for 5 days (D5). (F) Fluorescence staining and quantification for the measure of cellular ROS using DCFH in C2C12 myoblasts (scale bar = 100 μm). Data were expressed as the mean ± SD and statistically analysed via one‐ or two‐way ANOVA. **p < 0.05/**p < 0.01/***p < 0.001 versus Con group or 0 g/L group; ^&&^ *p* < 0.01/^&&&^ *p* < 0.001 versus 2 months group.

To investigate the role of ArfGAP3 in skeletal muscle ageing both in vivo and in vitro, we established D‐gal–induced ageing animal and cell models, following previously reported protocols [23]. Compared with the gradual increase in body weight of the control mice, the gain in the mice treated with D‐gal was slower (Figure S2A), and skeletal muscle mass decreased over time in both PFM (Figure 2A) and TA muscles (Figure S2B). Although there was no significant difference in MCC of mice (Figure S2C), the BLPP decreased after 4 months of D‐gal treatment (Figure 2B). We also observed a time‐related decrease of ArfGAP3 expression in PFM, accompanied by an increase in P16 and P53 expression following D‐gal treatment (Figure 2C). Moreover, D‐gal treatment also elicited oxidative stress, evidenced by elevated levels of MDA and reduced activity of CAT, GST and SOD (Figure S2D).

In the D‐gal–induced ageing C2C12 model, cellular senescence in C2C12 myoblasts worsened with increasing doses of D‐gal (Figure 2D, top panels). After 5 days of differentiation (D5), we observed a diminished myogenic differentiation capacity of C2C12 cells after D‐gal treatment, manifested by smaller myotube diameters and reduced myotube formation, as showed in Figre2D (middle panel). We also noted an increased presence of SA‐β‐gal‐positive myotubes in C2C12 myotubes treated with 40 g/L D‐gal (Figure 2D, lower panel). Similarly, the downregulation of *Arfgap3* mRNA during cellular senescence was also specific, compared with *Arfgap1* and *Arfgap2* (Figure S2E). ArfGAP3 was downregulated after D‐gal treatment, while the expression of P16 and p53 showed a concentration‐dependent increase (Figure 2E). D‐gal treatment also downregulated the expression of the myotube differentiation markers MyhC, MyoG and MyoD (Figure 2E). After D‐gal treatment, we observed a decrease in mitochondrial membrane potential (MMP) as indicated by JC‐1 staining (Figure S2F) and increased cellular reactive oxygen species (ROS) levels using DCFH‐DA probes (Figure 2F). We further established an aged C2C12 model through replicative senescence, which represents natural cellular ageing (Supporting Information S1, Supporting Information S5). As expected, aged C2C12 cells (Figure S3A) also exhibited decreased levels of ArfGAP3 (Figure S3B–C), diminished differentiation capacity (Figure S3D), increased expression of senescence‐associated proteins (Figure S3C) and reduced antioxidant defence (Figure S3E).

### 3.3 Downregulation of ArfGAP3 in C2C12 Myoblasts Impaired Differentiation, Proliferation Capacity and Mitochondrial Function

**FIGURE 3:**
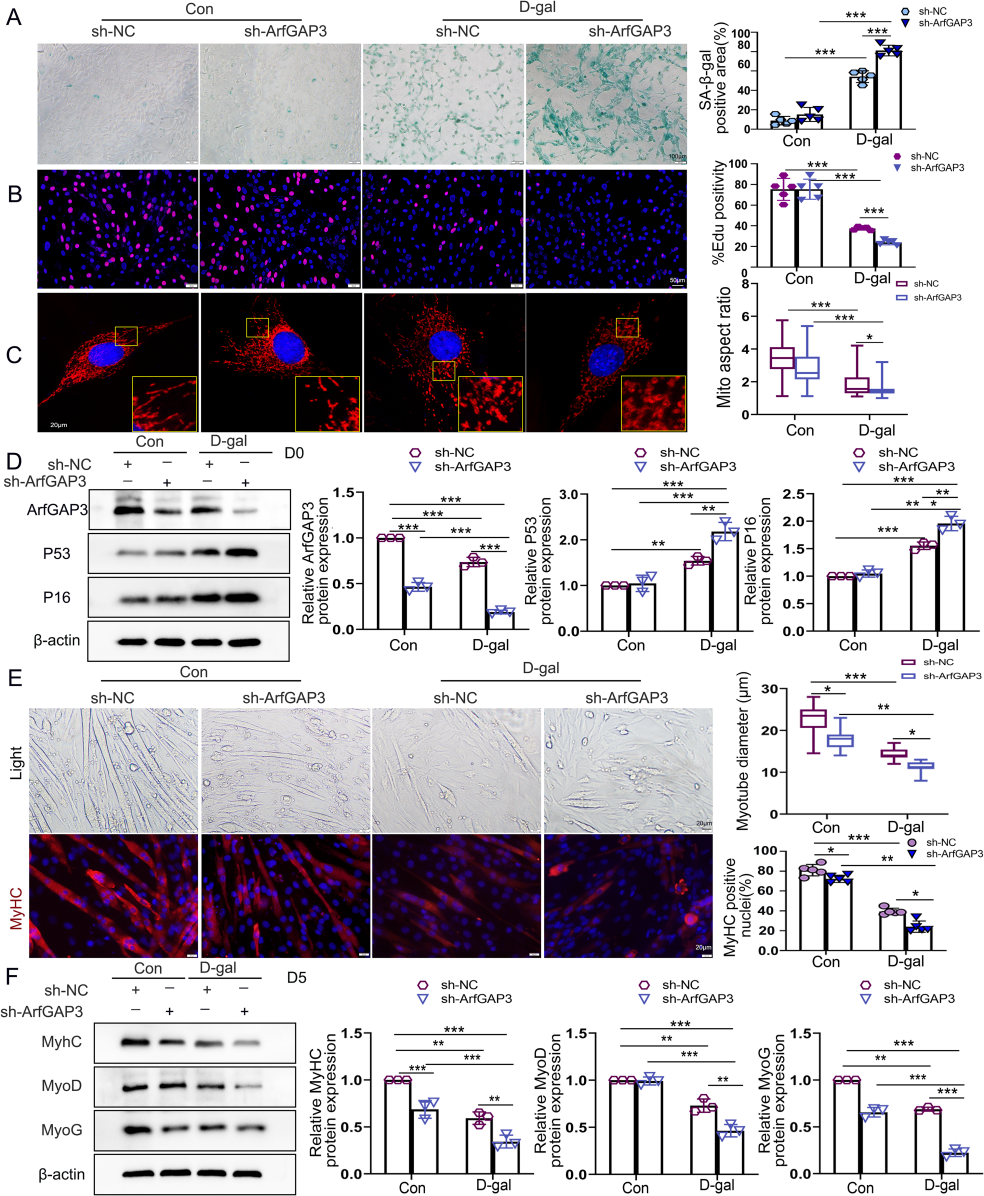
Downregulation of ArfGAP3 in C2C12 myoblasts impaired differentiation, proliferation capacity and mitochondrial function. (A) SA‐β‐gal staining and corresponding quantification in C2C12 myoblasts; scale bar = 100 μm. (B) Representative images from EdU staining and quantification of the relative percentages of EdU‐positive myoblasts in EdU assays after transfection with lentiviral vector with or without 20 g/L D‐gal treatment; scale bar = 50 μm. (C) Representative confocal microscopy images of MitoTracker (red) staining in C2C12 myoblasts. Scale bar = 20 μm; (D) Western blot analysis and quantification of ArfGAP3, P16 and P53 protein levels. (E) Microscopic visualization of myotube formation (upper panels) and representative immunofluorescence images of C2C12 myotubes with MyhC stain (bottom panels). Quantifications were for myotube diameter and the percentage of MyhC‐positive nuclei (differentiation index). Scale bar = 20 μm. (F) Western blot analysis and respective quantification for MyhC, MyoG and MyoD after differentiation for 5 days. All data were presented as mean ± SD. All analyses were conducted using two‐way ANOVA. *p < 0.05, **p < 0.01, ***p < 0.001.

We then investigated the role of ArfGAP3 in muscle ageing using targeted knockdown of ArfGAP3 (sh‐ArfGAP3) and applying D‐gal (20 g/L) treatment to simulate muscle ageing. The effectiveness of ArfGAP3 knockdown was confirmed by decreased mRNA levels, which were further reduced after D‐gal treatment (Figure S4A). Surprisingly, the level of cellular senescence was not induced by knocking down ArfGAP3, but exacerbated after D‐gal treatment (Figure 3A), indicating a similar effect on cell proliferation capacity (Figure 3B). Mitochondrial morphology showed no significant differences between the sh‐NC and sh‐ArfGAP3 groups, but D‐gal treatment resulted in fragmented and swollen mitochondria, particularly in the sh‐ArfGAP3 group (Figure 3C and Figure S4B). Furthermore, ArfGAP3 knockdown also worsened the decline in MMP in D‐gal–induced senescent C2C12 myoblasts (Figure S3C). The protein expression level of ArfGAP3 significantly decreased after lentivirus transfection, with further reduction after D‐gal treatment (Figure 3D). There were no differences in the expression of P16 and P53 between the sh‐NC and sh‐ArfGAP3 groups, but a higher upregulation of P16 and P53 protein expression was observed after D‐gal treatment in the sh‐ArfGAP3 group (Figure 3D). Despite no effects on cellular senescence or mitochondrial function, ArfGAP3 knockdown led to a simultaneous reduction in myotube differentiation diameter and differentiation index. Furthermore, D‐gal treatment exacerbated the impaired differentiation function after ArfGAP3 knockdown (Figure 3E). Interestingly, ArfGAP3 knockdown did not affect the expression of the early differentiation factor MyoD, but decreased the expression of late differentiation proteins MyoG and MyhC. These findings suggest that ArfGAP3 plays a crucial role in late‐stage differentiation during ageing.

### 3.4 Overexpression of ArfGAP3 Improved the Differentiation Capacity by Rescuing Mitochondrial Function in D‐Gal–Treated C2C12 Myoblasts

**FIGURE 4:**
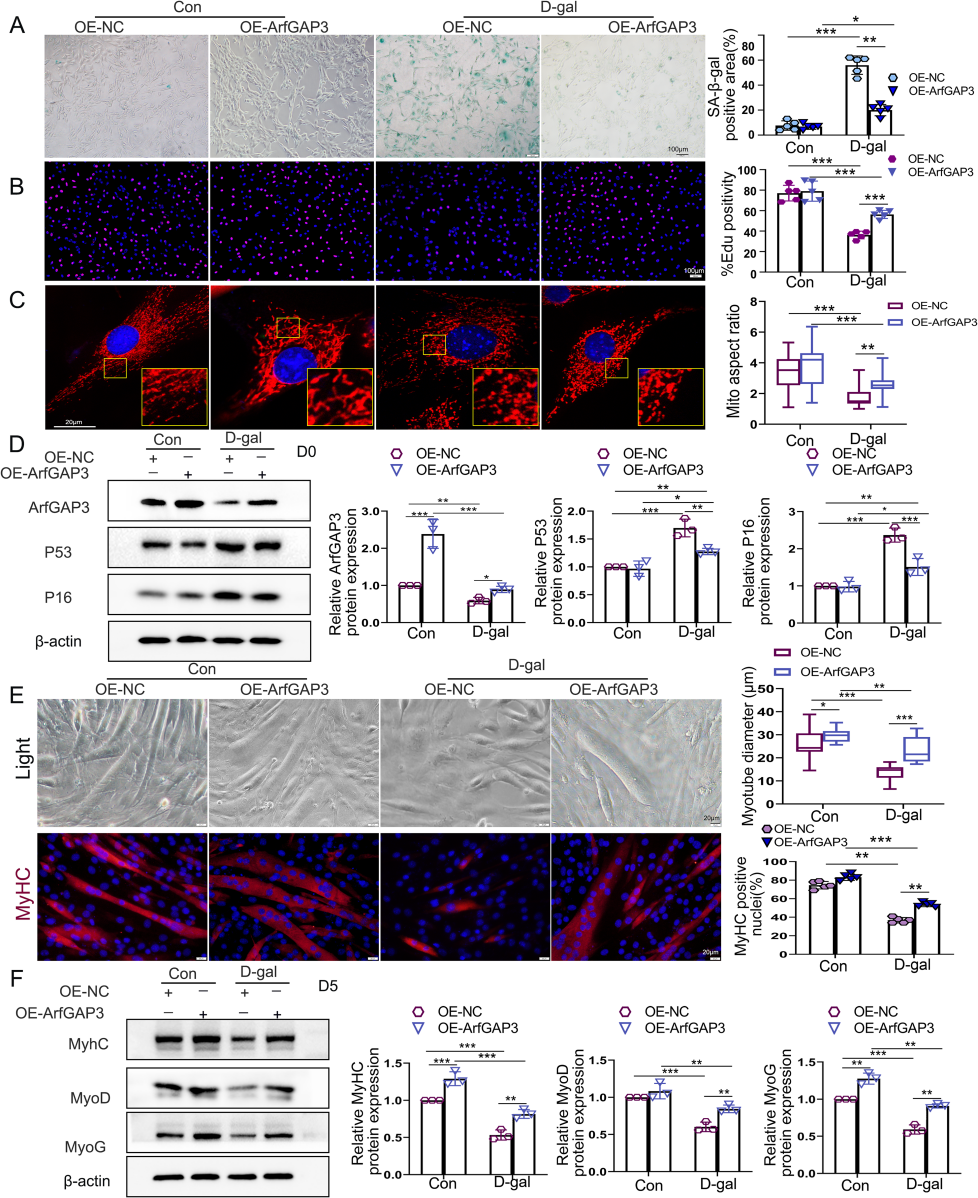
Overexpression of ArfGAP3 improved the differentiation capacity by rescuing mitochondrial function in D‐gal–treated C2C12 myoblasts. (A) SA‐β‐gal staining and quantification in C2C12 myoblasts; scale bar = 100 μm. (B) Representative images of EdU staining and quantification of the relative percentages of EdU‐positive myoblasts after transfection with lentiviral vector with or without 20 g/L D‐gal treatment; scale bar = 100 μm. (C) Representative confocal microscopy images of MitoTracker (red) staining in C2C12 myoblasts. Scale bar = 20 μm; (D) Western blot analysis and quantification of ArfGAP3, P16 and P53 protein levels. (E) Microscopic visualization of myotube formation (upper panels) and representative immunofluorescence images of C2C12 myotubes with MyhC stain (bottom panels). Quantifications were for myotube diameter and the percentage of MyhC‐positive nuclei (differentiation index). Scale bar = 20 μm. (F) Western blot analysis and respective quantification for MyhC, MyoG and MyoD after differentiation for 5 days. All data were presented as mean ± SD. All analyses were conducted using two‐way ANOVA. *p < 0.05, **p < 0.01, ***p < 0.001.

Next, to determine whether ArfGAP3 overexpression could reverse the impaired differentiation capacity of D‐gal–treated C2C12 myoblasts, we generated a C2C12 stable‐cell line overexpressing ArfGAP3 (OE‐ArfGAP3). ArfGAP3 overexpression was confirmed at mRNA level and protein level (Figure S5A and Figure 4D). Notably, we observed that cell senescence induced by D‐gal was alleviated by ArfGAP3 overexpression (Figure 4A). As expected, ArfGAP3 overexpression also rescued the impaired cell proliferation capacity (Figure 4B) as well as mitochondrial morphology and function, as evidenced by improvements in aspect ratio, mitochondrial length and MMP (Figure 4C and Figure S5B,C). The induced P16 and P53 upon D‐gal‐stimulation was reduced in OE‐ArfGAP3 group (Figure 4D). We further observed that the size of myotubes and fusion index were also increased by ArfGAP3 overexpression (Figure 4E). Consequently, the impaired differentiation capacity induced by D‐gal treatment was reversed in ArfGAP3 overexpressed cells (Figure 4E). Western blotting revealed that MyoG and MyhC protein levels were upregulated upon ArfGAP3 overexpression whereas no difference in MyoD expression was observed (Figure 4F). However, D‐gal–triggered reduction in MyoD, MyoG and MyhC protein levels were all reversed in ArfGAP3 overexpressed cells (Figure 4F). The above data implied that ArfGAP3 overexpression improved the differentiation capacity and rescued mitochondrial function in D‐gal–treated C2C12 myoblasts.

### 3.5 Rab5a Played a Critical Role in the Effect of ArfGAP3

**FIGURE 5:**
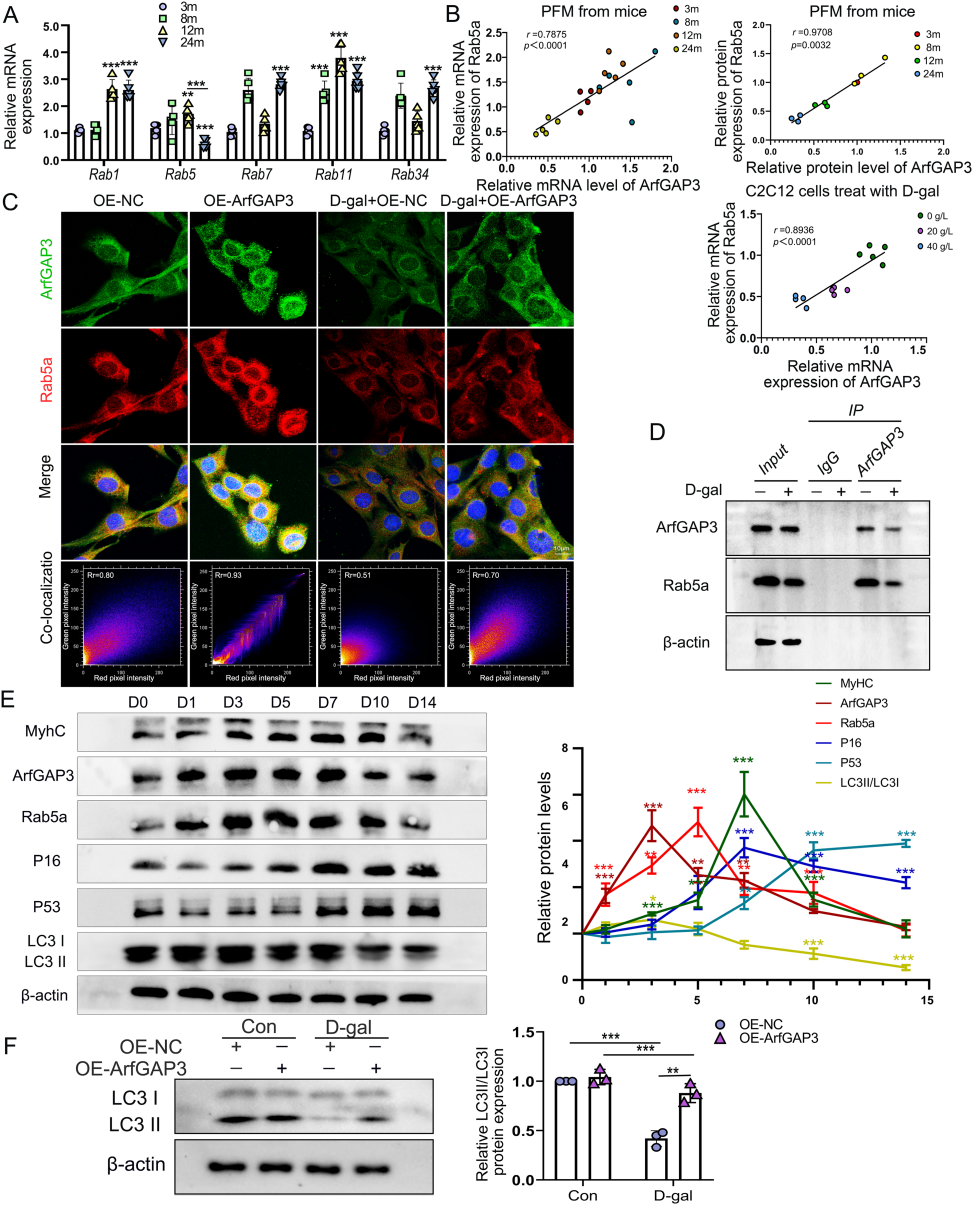
Rab5a played a critical role in the effect of ArfGAP3. (A) Comparison of relative mRNA expressions of Rab1, Rab5, Rab7, Rab11 and Rab34 in PFM of 3‐, 8‐, 12‐ and 24‐month‐old mice. (B) Correlation graphs between the mRNA and protein expression of *ArfGAP3* and *Rab5a* in PFM of 3‐, 8‐, 12‐ and 24‐month‐old mice and in C2C12 myoblasts treated with D‐gal. (C) Images of immunofluorescence staining to present spatial expression patterns of ArfGAP3 (red) and Rab5a (green) in C2C12 myoblasts after transfection. Scale bar = 10 μm. The colocalization scatter plot (bottom panels) represents fluorescence intensity profiles extracted for quantification. (D) Coimmunoprecipitation (CoIP) experiment using an antibody against ArfGAP3 to detect the interaction between ArfGAP3 and Rab5a in C2C12 myoblasts with or without 20 g/L D‐gal treatment, and the CoIP products were subjected to immunoblotting assay of ArfGAP3 and Rab5a. (E) Western blot analysis and quantification of the temporal expression of MyHC, ArfGAP3, Rab5a, P16, P53 and LC3 in C2C12 myoblasts during myotube differentiation from 0 (D0) to 14 days (D14). (F) Western blot analysis and quantification of LC3 II/LC3I ratio in C2C12 myoblasts after ArfGAP3 overexpression with or without 20 g/L D‐gal treatment. All data were presented as mean ± SD. All analyses were done using one‐ or two‐way ANOVA. *p < 0.05, **p < 0.01, ***p < 0.001.

ArfGAP3, known for its involvement in cargo sorting of ArfGAPs and colocalization with early endosomes, has garnered attention for its potential role in endosomal transport and autophagy regulation. Autophagy is important for maintaining stemness and preventing senescence during skeletal muscle ageing [24]. To investigate the involvement of Rab small GTPases, including Rab1, Rab5, Rab7, Rab11 and Rab34, in autophagy regulation, we examined their mRNA expression levels. Notably, we observed consistent trends in the expression of Rab5a (also known as Rab5) with ArfGAP3 (Figure 5A). Pearson correlation analysis revealed a significant positive correlation between ArfGAP3 and Rab5a mRNA expression in both aged mice and senescent C2C12 myoblasts, which was confirmed at the protein level (Figure 5B). Immunofluorescence staining showed decreased expression of both ArfGAP3 and Rab5a in aged C2C12 cells, accompanied by a decline in their expression correlation (Figure S6A). Overexpression of ArfGAP3 counteracted the downregulation of Rab5a induced by D‐galactose (D‐gal) and enhanced their colocalization (Figure 5C and Figure S6B). Coimmunoprecipitation assay demonstrated an interaction between ArfGAP3 and Rab5a, which decreased upon D‐gal treatment due to downregulation of ArfGAP3 (Figure 5D). During myotube differentiation, both ArfGAP3 and Rab5a exhibited elevated expression levels, followed by a gradual downregulation, paralleling the pattern of MyhC expression (Figure 5E). ArfGAP3 overexpression increased the abundance of the LC3 II isoform, suggesting enhanced autophagy flux (Figure 5F). Chloroquine (CQ) treatment, an inhibitor of lysosomes, induced impaired autophagosome‐lysosome fusion and LC3 II accumulation. However, ArfGAP3 knockdown resulted in a lower level of LC3 II, even in the presence of chloroquine (Figure S6C). Further inhibition of autophagosome precursors using 3‐methyladenine (3‐MA) confirmed the role of ArfGAP3 in autophagy regulation (Figure S6D). These findings suggest that the ArfGAP3‐mediated regulation of Rab5a may be a critical mechanism underlying autophagy during ageing.

### 3.6 Rab5a‐Mediated Autophagy Was Activated by the Regulatory Effect of ArfGAP3

**FIGURE 6:**
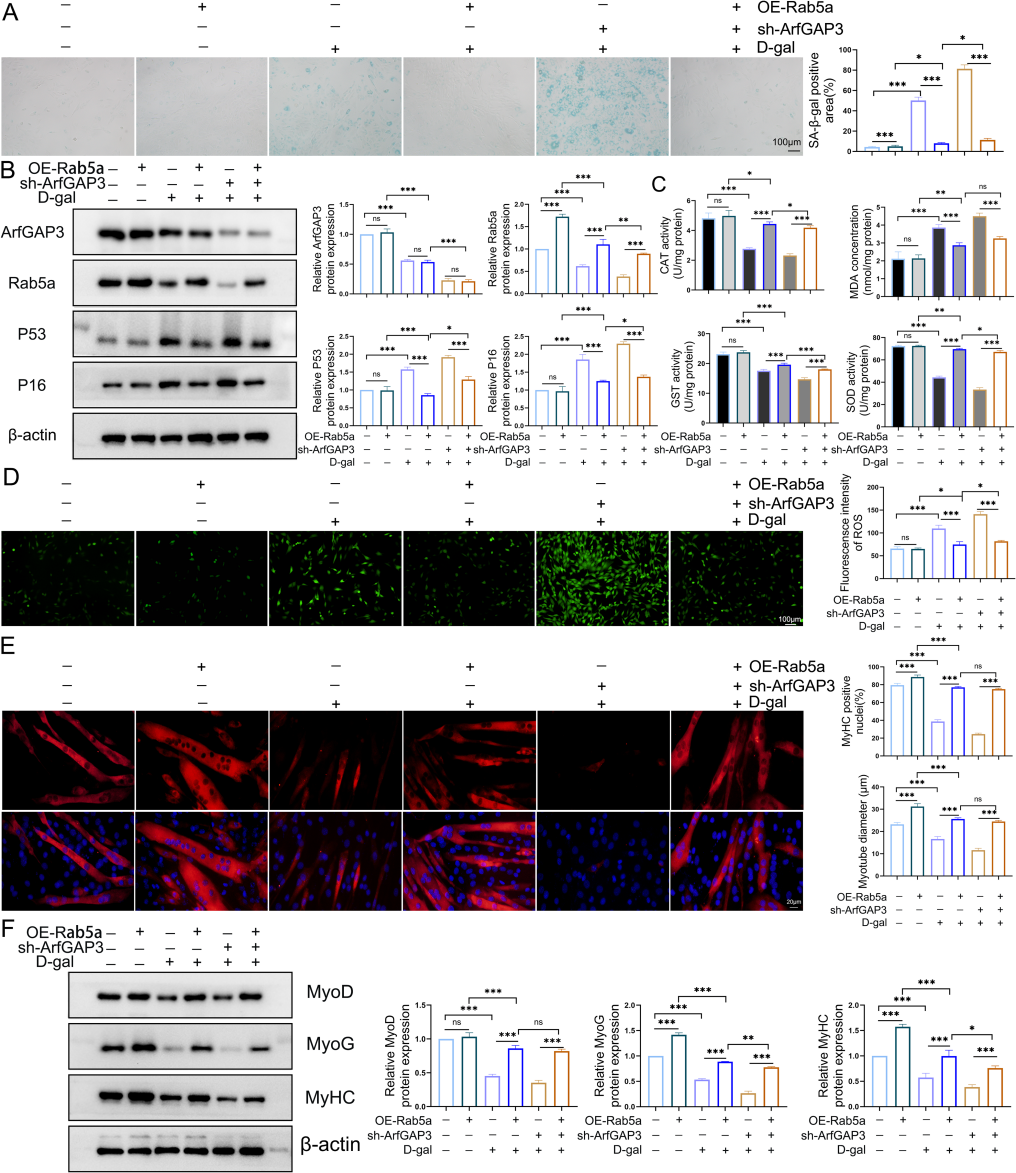
Rab5a‐mediated autophagy was activated in the regulatory effect of ArfGAP3. (A) SA‐β‐gal staining and quantification in C2C12 myoblasts with Rab5a overexpression in the presence of 20 g/L D‐gal; scale bar = 100 μm. (B) Western blot analysis and quantification of ArfGAP3, Rab5a, P16 and P53 proteins. (C) Contents of MDA in C2C12 cells and activities of antioxidant enzymes including CAT, GST and SOD. (D) ROS production evaluated by DCFH‐DA and ImageJ used for quantitative analysis. (E) Immunofluorescent staining for MyHC in myotubes after differentiation for 5 days and quantification for myotube diameter and the percentage of MyhC‐positive nuclei (differentiation index). Scale bar = 20 μm. (F) Western blot analysis and respective quantification for MyhC, MyoG and MyoD after differentiation for 5 days. Data were presented as mean ± SD. Statistical analyses were conducted using one‐way ANOVA. *p < 0.05, **p < 0.01, ***p < 0.001.

Autophagosome maturation involves the fusion of autophagosomes with early or late endosomes, with Rab5a playing a crucial role in promoting autophagosome formation. Next, we conducted rescue experiments by simultaneously expressing exogenous Rab5a alongside ArfGAP3 knockdown. Our findings demonstrated that D‐gal–induced C2C12 senescence was exacerbated by sh‐ArfGAP3 but rescued by Rab5a overexpression (Figure 6A). We showed that Rab5a overexpression downregulated the expression of P16 and P53 induced by D‐gal treatment without affecting ArfGAP3 expression (Figure 6B). Notably, antioxidant system assessment revealed that the concentration of MDA was increased by D‐gal and further elevated by sh‐ArfGAP3, whereas Rab5a overexpression restored these effects (Figure 6C). Correspondingly, a similar decrease in antioxidant enzyme activity induced by D‐gal including CAT, GST and SOD was reversed by Rab5a overexpression (Figure 6C). Consistent with the antioxidant system, ROS production induced by D‐gal treatment and exacerbated by sh‐ArfGAP3 was also ameliorated by Rab5a overexpression (Figure 6D) while the gene expression of *Nox2* and *Nox4* presented a similar profile (Figure S6F). Consistent with the previous findings [25], Rab5a overexpression increased myotube differentiation diameter and differentiation index, as well as myogenic markers MyhC and MyoG (Figure 6E,F). Moreover, Rab5a overexpression rescued myotube diameter and differentiation index reduced by D‐gal while increasing the level of myogenic markers including MyhC, MyoG and MyoD (Figure 6E,F).

**FIGURE 7:**
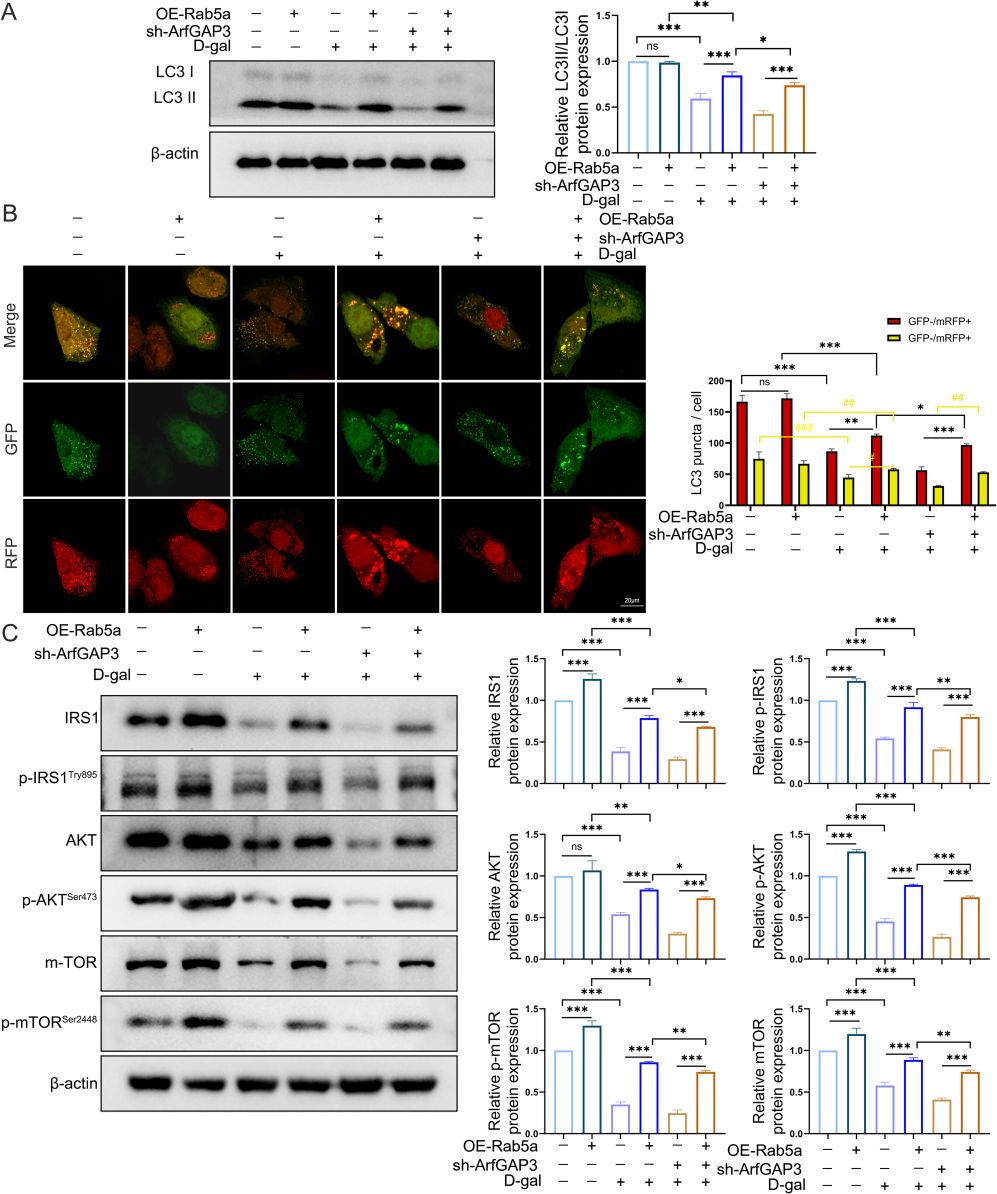
Rab5a overexpression rescued autophagy and activated signalling IRS1‐AKT‐mTOR pathway. C2C12 transfected with sh‐ArfGAP3 or/and Rab5a‐overexpression plasmid, followed by treatment with or without 20 g/L D‐gal. (A) Western blot analysis of LC3 and associated quantification. (B) Representative phase contrast, fluorescence photomicrographs and the quantification of GFP‐RFPLC3 puncta in C2C12 myoblasts were shown. Yellow puncta denote autophagic vesicle structures. The scale bars represent 20 μm. (C) Protein levels of Rab5a‐activated IRS1‐AKT‐mTOR signalling by western blotting analysis. All data were presented as mean ± SD. All analyses were done using one‐way ANOVA. *p < 0.05, **p < 0.01, ***p < 0.001.

The aforementioned findings have raised questions regarding the necessity of Rab5a expression in the regulation of autophagy. Thus, we evaluated the autophagy level by examining the LC3 II/LC3I ratio of protein in C2C12 myoblasts from the different experimental groups. The results showed D‐gal treatment induced decreased LC3 II/LC3I ratio, which was exacerbated by ArfGAP3 knockdown, however rescued by overexpression Rab5a (Figure 7A). To further assess the effect of ArfGAP3 on autophagic flux, the above C2C12 myoblasts were transduced with Ad‐mRFP‐GFP (the adenovirus‐mediated expression of mRFP and green fluorescent protein)‐LC3, a specific marker of autophagosome formation that relies on the different nature of GFP and RFP fluorescence under acidic conditions. Likewise, D‐gal–treated sh‐ArfGAP3 C2C12 myoblasts displayed minimal red‐only LC3‐II puncta, while Rab5a overexpression induced the formation of yellow LC3‐II puncta formation (Figure 7B). Concerning the pivotal role of Rab5a as a critical switch controlling AKT‐mTOR signalling by activating IRS1 in promoting myogenesis [25], we also revealed that the expression levels of p‐IRS1, p‐AKT and p‐mTOR were markedly upregulated after Rab5a overexpression. The IRS1‐AKT‐mTOR signalling related proteins reduced upon D‐gal treatment while further exacerbated in the sh‐ArfGAP3 group after differentiation for 5 days, which were reverses by overexpression Rab5a (Figure 7C). To reinforce the regulatory mechanism between ArfGAP3 and Rab5a, we further knockdown Rab5a with siRNA (si‐Rab5a) in C2C12 cells. Knockdown of Rab5a exacerbated cellular senescence (Figure S7B), while weakening the rescue effect of ArfGAP3 overexpression in ageing including autophagic flux (Figure S7A), antioxidant capacity (Figure S7C) and myogenic differentiation capacity (Figure S7D). Additionally, the increased levels of p‐IRS1, p‐AKT and p‐mTOR by ArfGAP3 overexpression was also abolished by Rab5a knockdown (Figure S7E). Taken together, these results suggested that ArfGAP3 enhanced differentiation capacity through Rab5a‐mediated IRS1‐AKT‐mTOR signalling and that Rab5a activation was involved in ArfGAP3‐promoted autophagic flux.

### 3.7 ArfGAP3 Overexpression Improved Muscle Mass and Promoted the Antioxidant Response and Autophagy in Ageing Mice

**FIGURE 8:**
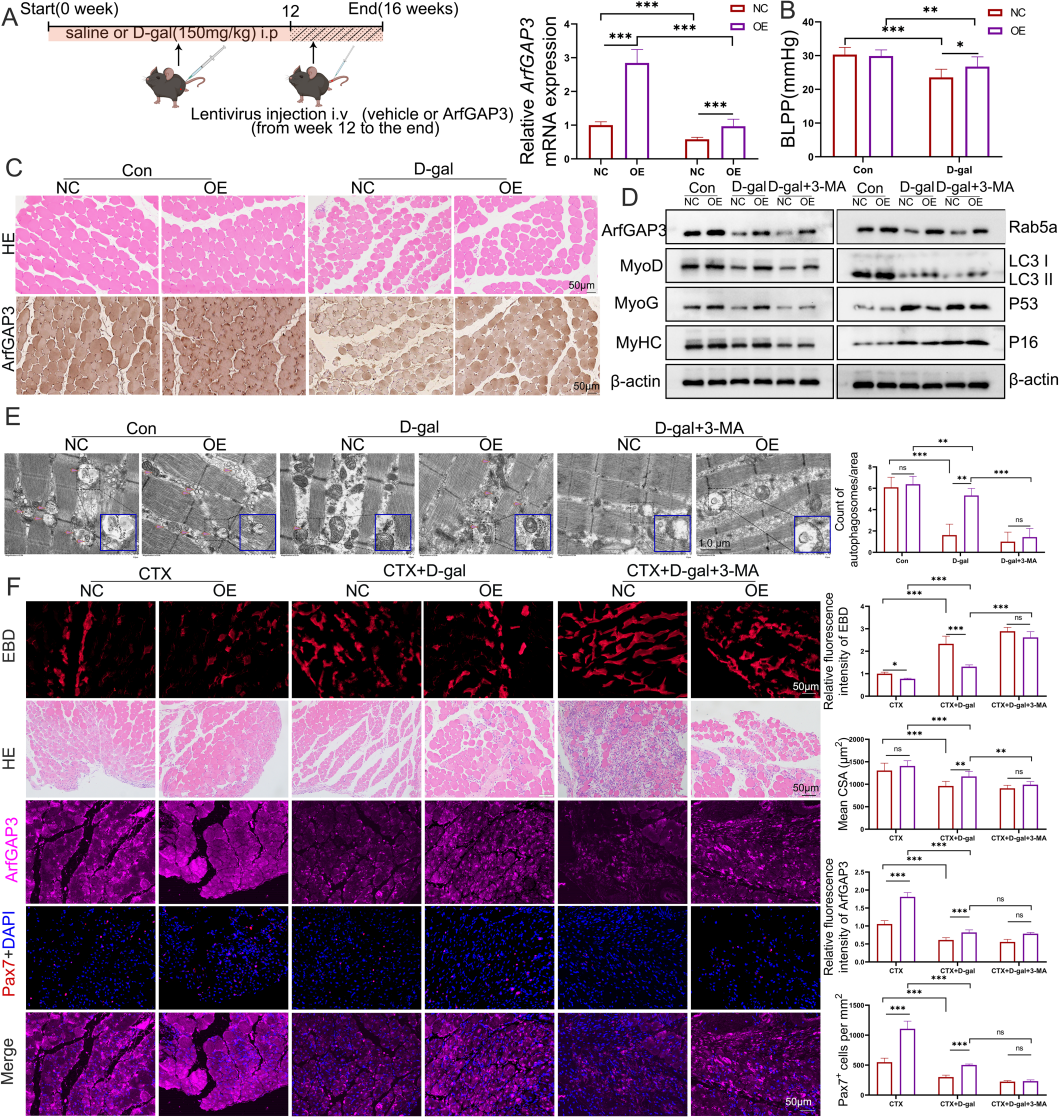
ArfGAP3 overexpression improved muscle mass and promoted the antioxidant response and autophagy in ageing mice. (A) Schematic diagram of the mouse experiment and the mRNA expression levels of ArfGAP3 were validated using qRT‐PCR. (B) The average bladder leak point pressure (BLPP) values from the mice. (C) H&E staining of PFM muscle fibres (magnification 200×, scale bar = 50 μm) and the CSA of muscle fibres measured by ImageJ software (upper panels). IHC images and quantification for ArfGAP3 in mouse PFM muscle (magnification 200×; scale bar = 50 μm; bottom panels). (D) Western blotting showing the protein levels of ArfGAP3, Rab5a, myogenic markers (MyHC, MyoD and MyoG), ageing‐related markers (P16 and P53) and autophagy‐related proteins (LC3). β‐Actin was used as the loading controls to quantify the relative protein levels. (E) Representative mitochondria were observed via transmission electron micrographs (magnification 12 000×) and quantification of the mean number of autophagosomes per area. Scale bar = 1.0 μm. (F) Representative images for Evans blue dye (EBD) assay, HE staining and immunofluorescence staining of PFM after 3 days of CTX injection in different groups were shown for the muscle histology. Scale bar = 50 μm. Quantification of EBD fluorescence intensity (red), CSA of centralized nuclear muscle fibres (μm^2^) and immunofluorescence intensities of ArfGAP3 and Pax 7 were showed on the right. The data are expressed as the mean ± SD and were analysed using one‐way ANOVA, ns, no significance, *p < 0.05, **p < 0.01 and ***p < 0.001; *n* = 5 mice per group.

ArfGAP3 overexpression in vivo was achieved through tail vein injection of lentiviruses in mice (Figure 8A), resulting in an increase in body weight and improved PFM function (indicated by the elevation of BLPP) (Figure S8A,C), without affecting MCC (Figure S8B). The rescuing effect of ArfGAP3 overexpression on muscle mass was also noted in TA (+9.8%), GA (+19.56%) and SOL (+11.25%) muscles in ageing models, while most pronounced in PFM (+25.31%) (Figure S8C). In addition, ArfGAP3 overexpression induced a slight, non‐significant increase in CSA of PFM in vivo, whereas significantly increased CSA of PFM in D‐gal treatment (Figure 8C top panels and Figure S8D). IHC staining confirmed a decreased expression of ArfGAP3 following D‐gal treatment, subsequently restored by overexpressing ArfGAP3 in PFM (Figure 8C bottom panels and Figure S8E). Furthermore, we noticed that neither BLPP nor muscle mass was increased by ArfGAP3 overexpression in the ageing PFM when treated with 3‐MA (Figure S9A,B). Western blot analysis demonstrated elevated ArfGAP3 protein levels in PFM through lentiviral overexpression, accompanied by increased Rab5a expressions and myogenic differentiation proteins of MyhC and MyoG in both basal and ageing mouse models. Additionally, increased expressions of MyoD and LC3II induced by ArfGAP3 overexpression were observed only in ageing PFM, while the upregulation of P16 and P53 induced by ageing was downregulated by ArfGAP3 overexpression. However, these differences induced by ArfGAP3 overexpression were abolished with 3‐MA treatment in ageing PFM (Figure 8D and Figure S9C). Consistently, ArfGAP3 overexpression alleviated mitochondrial damage and increased autophagosome formation in ageing PFM. However, these rescue effects were abolished by 3‐MA treatment (Figure 8E and Figure S9D). We further assessed the antioxidant system and found that ArfGAP3 overexpression decreased MDA production and enhanced the activity of antioxidant enzymes, including CAT, GST and SOD (Figure S9E). Nevertheless, these protective effects were also nullified by the 3‐MA treatment.

Our previous investigations revealed a significant upregulation of ArfGAP3 expression following mechanical injury to the PFM [21]. We hypothesized that this upregulation serves to facilitate the repair of PFM damage. Therefore, we conducted injections of cardiotoxin (CTX) into the PFM to elucidate the impact of ArfGAP3 on muscle regeneration during ageing and the Evans blue dye (EBD) assay was employed to assess muscle myofibre damage. Results indicated lower EBD fluorescence intensity induced by CTX in the ArfGAP3‐overexpressed PFM. D‐gal treatment induced a higher level of EBD fluorescence intensity, which was reversed by overexpression of ArfGAP3. However, 3‐MA treatment not only abrogated this effect but also further increased EBD fluorescence levels (Figure 8F). Histological analysis revealed a nonsignificant increase in regenerated muscle area in ArfGAP3‐overexpressed PFM. D‐gal treatment significantly reduced the CSA of regenerated muscle fibre, which was mitigated by ArfGAP3 overexpression. In contrast, PFM treated with 3‐MA showed almost no newly regenerated myofibres. Immunofluorescence results confirmed a positive correlation between ArfGAP3 expression levels and skeletal muscle regeneration levels. Specifically, we observed a significant reduction in Pax7^+^ cells in PFM due to D‐gal treatment, while the overexpression of ArfGAP3 increased Pax7^+^ cells and in both basal and ageing PFM. However, the inducible effect of ArfGAP3 overexpression was extinguished by 3‐MA treatment.

Overall, we have provided multiple lines of evidence showing that ArfGAP3 downregulation reduces the anti‐ageing capacity of the PFM, including impaired autophagy, weakened muscle regeneration capacity and reduced antioxidant capacity.

## 4 Discussion

Age‐related loss of muscle mass and function significantly impacts the quality of life in elderly individuals, increasing the risk of PFDs [26]. This is consistent with epidemiological findings [3, 27, 28] showing that the proportion of women with one or more PFDs dramatically increased from 6.3% in women aged 20–29 to 31.6% in women aged 50–59 years to 52.7% in women 80 and older. Therefore, we aimed to explore a potential target of an innovative pharmacological strategy to prevent age‐associated muscle weakness and restore physical autonomy in patients with PFDs. In our study, we observed a decrease in PFM mass in 24‐month‐old mice, equivalent to humans over 65 years old, suggesting that PFM undergo similar pathological changes as in humans. Additionally, PFM, anatomically weaker than hindlimb muscles due to lower basal muscle mass, displayed more pronounced ageing‐related effects, with a greater proportion of PFM mass loss. General pathological changes in aged skeletal muscle, including increased oxidative stress and decreased autophagy, were also observed in PFM. Therefore, investigating prevention and treatment strategies for PFDs based on the mechanisms of ageing‐related skeletal muscle loss holds promise.

ArfGAP3 plays an important role in cargo sorting in vesicle trafficking of the post‐Golgi membrane compartment [14]. Vesicular trafficking and membrane dynamics functions regulate transcriptional and posttranscriptional networks in adult muscle tissue homeostasis [29]. One possible explanation might be that ArfGAP3 was upregulated to promote cell differentiation and tissue repair in an acute injury of PFM induced by mechanical trauma, such as childbirth, and then was inhibited by various factors during the ageing process, resulting in an impaired capacity for differentiation and loss of skeletal muscle. Moreover, increasing evidence has identified the presence of giant mitochondria with a low inner mitochondrial membrane potential in aged muscle [30, 31], indicating that there may be multiple mechanisms at play in the regulation of mitochondrial morphology in aged skeletal muscle. Here, we observed large, misshapen mitochondria with disorganized cristae in aged PFM and showed that ArfGAP3 alleviated mitochondrial dysfunction by supporting the maintenance of normal mitochondrial morphology in the D‐gal–induced ageing process (Figure 3). We presumed ArfGAP3 improved skeletal muscle mass during ageing through a mechanism that maintains morphology.

Autophagy declines with age and impaired autophagy predisposes individuals to age‐related diseases, whereas interventions that stimulate autophagy often promote longevity [32]. Abnormal mitochondria accumulate within aged or diseased postmitotic cells as a consequence of insufficient autophagy, which is normally responsible for mitochondrial degradation [33]. Similarly, autophagic flux is important for preserving muscle mass and maintaining myofibre integrity [34]. Rab5a is indispensable at early endosomes, where it has a role in the activation of VPS34 [35] and promoting autophagosome formation [16]. Given that ArfGAP3 colocalized the most with Rab5a in endosomal transport, we found that ArfGAP3 influenced the expression of Rab5a and the IRS1‐IGF1‐AKT signalling pathway during C2C12 differentiation and regulated autophagic flux in the ageing process. Meanwhile, our in vivo study results also confirmed that overexpression of ArfGAP3 rescued muscle fibre area and BLPP in an ageing model, thereby enhancing anti‐ageing capabilities of PFM, while autophagy inhibitors abolish this protective effect (Figure 8). Therefore, it is reasonable to infer that the restoration of ArfGAP3 has an activating effect on mitochondrial function and autophagy to promote skeletal muscle regeneration in ageing skeletal muscle.

There are several limitations to our study that need to be acknowledged. Skeletal muscle regeneration is a complex process involving multiple factors that are sequentially activated to maintain muscle structure and function after injury. The specific causes of downregulated ArfGAP3 in the skeletal muscle of ageing mice remain largely unknown, and the mechanism by which ArfGAP3 regulates mitochondrial morphology has not been further elucidated, although we have found a possible involvement of autophagic flux. Additionally, early studies have identified the involvement of ARFGAP3 in follicular growth and oocyte maturation (Supporting Information S8), modulated by androgen [33]. It was also reported that the level of RAB5A mRNA was lower in obese patients with polycystic ovary syndrome and RAB5A regulated the follicle‐stimulating hormone (FSH)‐mediated FSH receptor (FSHR) signalling pathway [36]. Considering menopause as another significant factor contributing to pelvic floor dysfunction, we hypothesize that alterations in endocrine hormone levels (such as testosterone, FSH and oestrogen) during ageing are likely to be responsible for the decline in ArfGAP3 expression, but further research is needed to investigate this hypothesis.

## 5 Conclusion

In summary, our study represents the first investigation into the expression of ArfGAP3 in ageing‐related processes and provides evidence supporting the notion that ArfGAP3 promotes skeletal muscle function during ageing through the promotion of autophagic flux mediated by Rab5a. These findings highlight ArfGAP3 as a potential therapeutic target for mitigating the loss of skeletal muscle function in ageing.

## Conflicts of Interest

The authors declare no conflicts of interest.

## Supporting information


**Figure S1** (A) Comparison analysis of ArfGAP3 expression in 53 young, 73 healthy older and 61 frail older subjects in GSE117525. (B) The curves for body weight of 3‐, 8‐, 12‐ and 24‐month‐old mice (*n* = 15). (C) Average maximum cystometric capacity (MCC) of mice in the four groups (n = 15). (D) The mRNA levels of the oxidative stress‐related genes (*Nox2* and *Nox4*) by qRT‐PCR. (E) Assessments of MDA contents in C2C12 cells and activities of antioxidant enzymes including CAT, GST, and SOD. (F) Quantification for the count of damaged and normal mitochondria in PFM from TEM analysis. All data were presented as mean ± SD (*n* = 3). Data were analysed using one‐way ANOVA. *p < 0.05 / **p < 0.01 / ***p < 0.001 vs. 3 mon group; & < 0.05 /&& < 0.01 / &&&p < 0.001 vs.8 mon group; #p < 0.05/ ## < 0.01/ ### p < 0.05 vs. 12 mon group.

**Figure S2** (A) Body weight curves from control (Con) and D‐gal‐treated (D‐gal) mice (*n* = 15). (B) Weights assessment of Anterior (TA) muscle (n = 15). (C) Average maximum cystometric capacity (MCC) of mice (n = 15). (D) Assessments of MDA contents in C2C12 cells and activities of antioxidant enzymes including CAT, GST, and SOD. (E) qRT‐PCR analysis of the expression of *Arfgap1, Arfgap2* and *Arfgap3*. *β‐actin* was used as the loading control (*n* = 3). (F) Representative fluorescence images of MMP and quantification after incubation with JC‐1 in C2C12 myoblasts. Red fluorescence represents JC‐1 aggregates in healthy mitochondria, whereas green fluorescence represents JC‐1 monomers, indicating MMP dissipation (*n* = 5). Merged images represent colocalization of the JC‐1 aggregates and JC‐1 monomers (scale bar = 50 μm). Data were expressed as the mean ± SD, and analysed using one‐way ANOVA. **p < 0.05/ **p < 0.01 / ***p < 0.001 vs. Con group or 0 g/L group; &&p < 0.01/ &&&p < 0.001 vs. 2 months group.

**Figure S3** (A) SA‐β‐gal staining and quantification for young and aged C2C12 cells of a model of multiple population doublings. Scale bar = 100 μm. (B) qRT‐PCR analysis of the expression of Arfgap1, Arfgap2 and Arfgap3. β‐actin was used as the loading control. (C) Western blot analysis and quantification of ArfGAP3, P16 and P53 proteins in young and aged C2C12 cells (D0) and protein levels of MyHC, MyoG and MyoD after differentiation for 5 days (D5). (D) Immunofluorescent staining for MyHC in young and aged C2C12 cells after differentiation for 5 days and quantification for myotube diameter and the percentage of MyhC‐positive nuclei (differentiation index). Scale bar = 20 μm. (E) Assessments of MDA contents in C2C12 cells and activities of antioxidant enzymes including CAT, GST, and SOD. Data were expressed as the mean ± SD, and an unpaired two‐tailed Student’s t test was used to analyse the statistical significance between two groups. *p < 0.05, **p < 0.01, ***p < 0.001.

**Figure S4** (A) The mRNA level of ArfGAP3 in C2C12 myoblasts. (B) Representative immunofluorescence micrographs transferred to 8 bits images for quantification of C2C12 myoblasts. (C) Representative fluorescence images of MMP and quantification after incubation with JC‐1 in C2C12 myoblasts. Data were expressed as the mean ± SD and analysed using one‐ or two‐way ANOVA. **p < 0.01/ ***p < 0.001 vs. Con group.

**Figure S5** (A) The mRNA levels of ArfGAP3 in C2C12 myoblasts after plasmid transfection with or without D‐gal treatment for 48 h. (B) Representative immunofluorescence micrographs transferred to 8 bits images for quantification of C2C12 myoblasts. (C) Representative fluorescence images of MMP and quantification after incubation with JC‐1 in C2C12 myoblasts. Data were expressed as the mean ± SD and analysed using one‐ or two‐way ANOVA. **p < 0.01/ ***p < 0.001 vs. Con group.

**Figure S6** (A) Images of immunofluorescence staining to present spatial expression patterns of ArfGAP3 (red) and Rab5a (green) in young and aged C2C12 cells. Scale bar = 20 μm. (B) The co‐localization curves that represented fluorescence intensity profiles were calculated from images. (C) Western blot analysis of LC3 proteins in C2C12 myoblasts treated with 10 mM chloroquine (CQ) and 2 mM 3‐methyladenine (3‐MA) (D) after ArfGAP3 knockdown. (E) The mRNA levels of *Nox2* and *Nox4*. All data were presented as mean ± SD. All analyses were done using one‐ or two‐way ANOVA. *p < 0.05, **p < 0.01, ***p < 0.001.

**Figure S7** (A) Western blot analysis and quantification of ArfGAP3, Rab5a and LC3 II/LC3I ratio levels. (B) SA‐β‐gal staining and quantification in C2C12 myoblasts with Rab5a knockdown in the presence of 20 g/L D‐gal; scale bar = 100 μm. (C) Contents of MDA in C2C12 cells and activities of antioxidant enzymes including CAT, GST, and SOD. (D) Immunofluorescent staining for MyHC in myotubes after differentiation for 5 days and quantification for myotube diameter and the percentage of MyhC‐positive nuclei (differentiation index). Scale bar = 20 μm. (F) Western blot analysis and respective quantification for MyhC, MyoG and MyoD after differentiation for 5 days. Data were presented as mean ± SD. Statistical analyses were conducted using one‐way ANOVA. *p < 0.05, **p < 0.01, ***p < 0.001.

**Figure S8** (A) Body weight of mice in NC and OE group with or without D‐gal treatment for 4 months. (B) Average maximum cystometric capacity (MCC) of mice. (C) Wet weights of TA, GA, SOL and PFM muscles in NC and OE group with or without D‐gal treatment for 4 months. (D) The CSA of muscle fibres measured by ImageJ software. (E) The quantification for ArfGAP3 from IHC images in mouse PFM muscle (magnification 200×; scale bar = 50 μm; (bottom panels).

**Figure S9** (A) The average BLPP and MCC(B) values of mice. (C) Wet weights of PFM, TA, GA and SOL muscles. (PFM: pelvic floor muscle, GA: gastrocnemius, TA: tibialis anterior, and SOL: soleus) (F) Quantification of the count of damaged and normal mitochondria in PFM of TEM analysis. (G) Assessments for MDA content and the activity of antioxidant enzymes, including CAT, GST and SOD. All data were presented as mean ± SD. All analyses were done using one‐ or two‐way ANOVA. *p < 0.05, **p < 0.01, ***p < 0.001.

**Table S1** Primer sequences for mouse genes.
