# Supplementary figures and images for: ArfGAP3 Protects Mitochondrial Function and Promotes Autophagy Through Rab5a‐Mediated Signals in Ageing Skeletal Muscle
Source: J Cachexia Sarcopenia Muscle. 2025 Feb 17;16(1):e13725. doi: 10.1002/jcsm.13725 (PMC11832210; doi:10.1002/jcsm.13725)

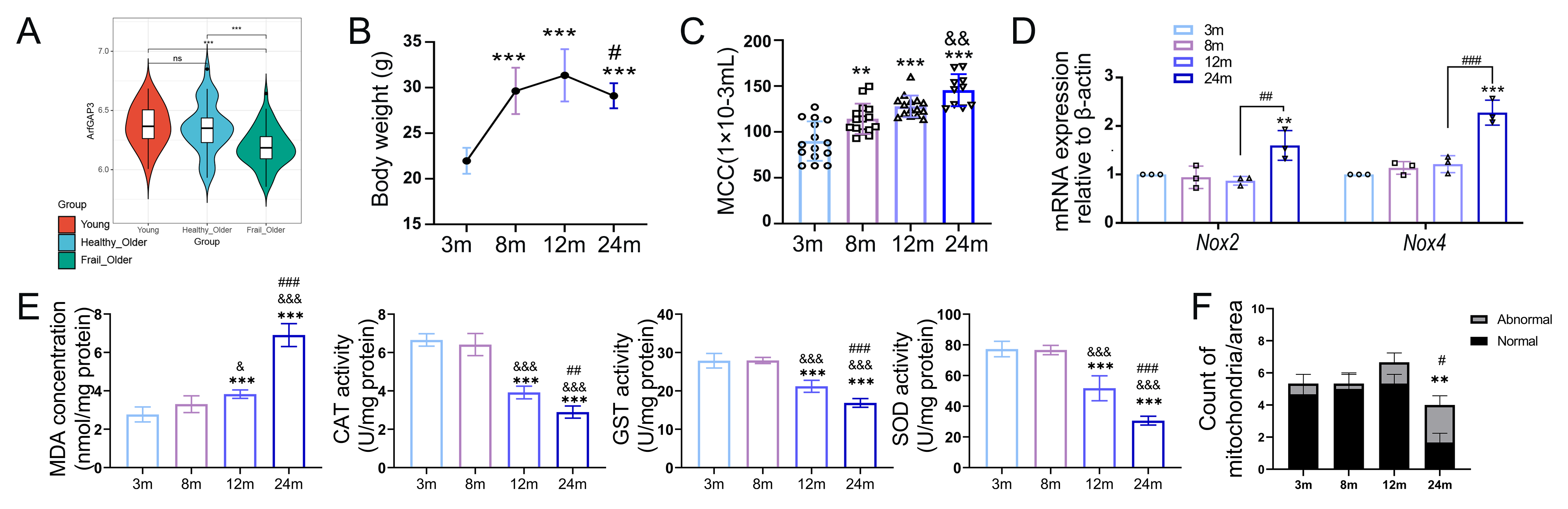

Supplement: Supplementary file 1 — Figure S1 (A) Comparison analysis of ArfGAP3 expression in 53 young, 73 healthy older and 61 frail older subjects in GSE117525. (B) The curves for body weight of 3‐, 8‐, 12‐ and 24‐month‐old mice (n = 15). (C) Average maximum cystometric capacity (MCC) of mice in the four groups (n = 15). (D) The mRNA levels of the oxidative stress‐related genes (Nox2 and Nox4) by qRT‐PCR. (E) Assessments of MDA contents in C2C12 cells and activities of antioxidant enzymes including CAT, GST, and SOD. (F) Quantification for the count of damaged and normal mitochondria in PFM from TEM analysis. All data were presented as mean ± SD (n = 3). Data were analysed using one‐way ANOVA. *p < 0.05 / **p < 0.01 / ***p < 0.001 vs. 3 mon group; & < 0.05 /&& < 0.01 / &&&p < 0.001 vs.8 mon group; #p < 0.05/ ## < 0.01/ ### p < 0.05 vs. 12 mon group. [file JCSM-16-e13725-s008.tif]

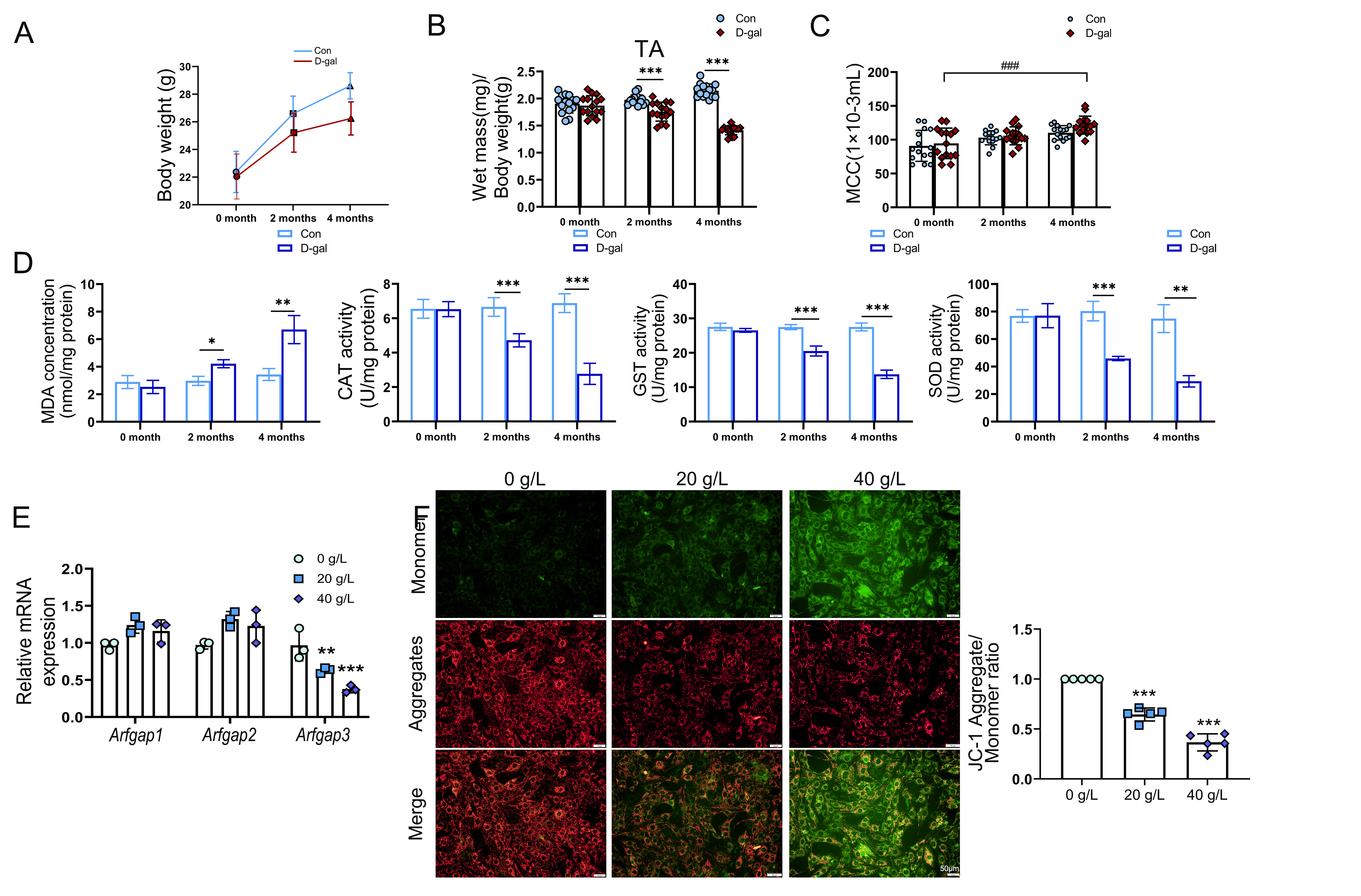

Supplement: Supplementary file 2 — Figure S2 (A) Body weight curves from control (Con) and D‐gal‐treated (D‐gal) mice (n = 15). (B) Weights assessment of Anterior (TA) muscle (n = 15). (C) Average maximum cystometric capacity (MCC) of mice (n = 15). (D) Assessments of MDA contents in C2C12 cells and activities of antioxidant enzymes including CAT, GST, and SOD. (E) qRT‐PCR analysis of the expression of Arfgap1, Arfgap2 and Arfgap3. β‐actin was used as the loading control (n = 3). (F) Representative fluorescence images of MMP and quantification after incubation with JC‐1 in C2C12 myoblasts. Red fluorescence represents JC‐1 aggregates in healthy mitochondria, whereas green fluorescence represents JC‐1 monomers, indicating MMP dissipation (n = 5). Merged images represent colocalization of the JC‐1 aggregates and JC‐1 monomers (scale bar = 50 μm). Data were expressed as the mean ± SD, and analysed using one‐way ANOVA. **p < 0.05/ **p < 0.01 / ***p < 0.001 vs. Con group or 0 g/L group; &&p < 0.01/ &&&p < 0.001 vs. 2 months group. [file JCSM-16-e13725-s007.tif]

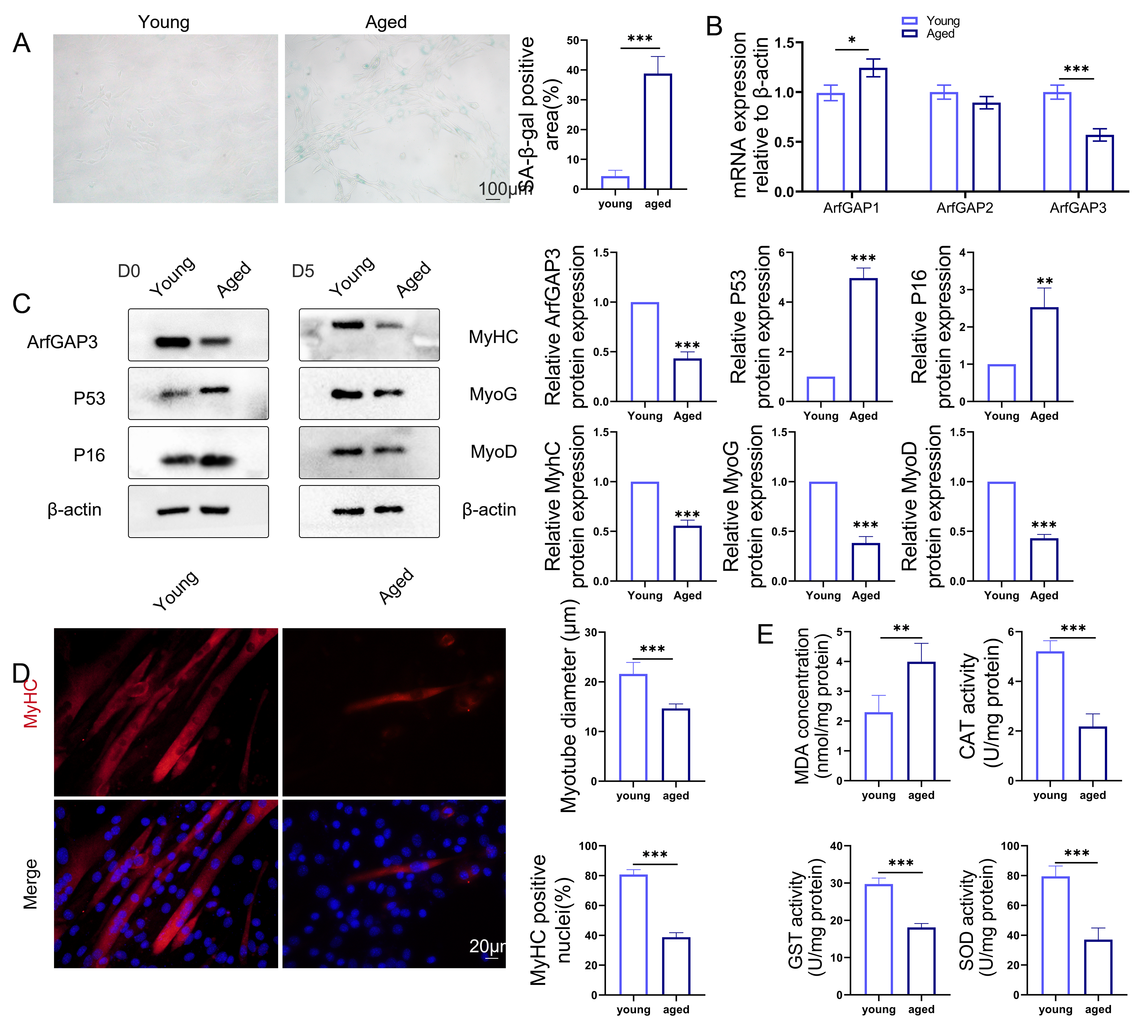

Supplement: Supplementary file 3 — Figure S3 (A) SA‐β‐gal staining and quantification for young and aged C2C12 cells of a model of multiple population doublings. Scale bar = 100 μm. (B) qRT‐PCR analysis of the expression of Arfgap1, Arfgap2 and Arfgap3. β‐actin was used as the loading control. (C) Western blot analysis and quantification of ArfGAP3, P16 and P53 proteins in young and aged C2C12 cells (D0) and protein levels of MyHC, MyoG and MyoD after differentiation for 5 days (D5). (D) Immunofluorescent staining for MyHC in young and aged C2C12 cells after differentiation for 5 days and quantification for myotube diameter and the percentage of MyhC‐positive nuclei (differentiation index). Scale bar = 20 μm. (E) Assessments of MDA contents in C2C12 cells and activities of antioxidant enzymes including CAT, GST, and SOD. Data were expressed as the mean ± SD, and an unpaired two‐tailed Student’s t test was used to analyse the statistical significance between two groups. *p < 0.05, **p < 0.01, ***p < 0.001. [file JCSM-16-e13725-s009.tif]

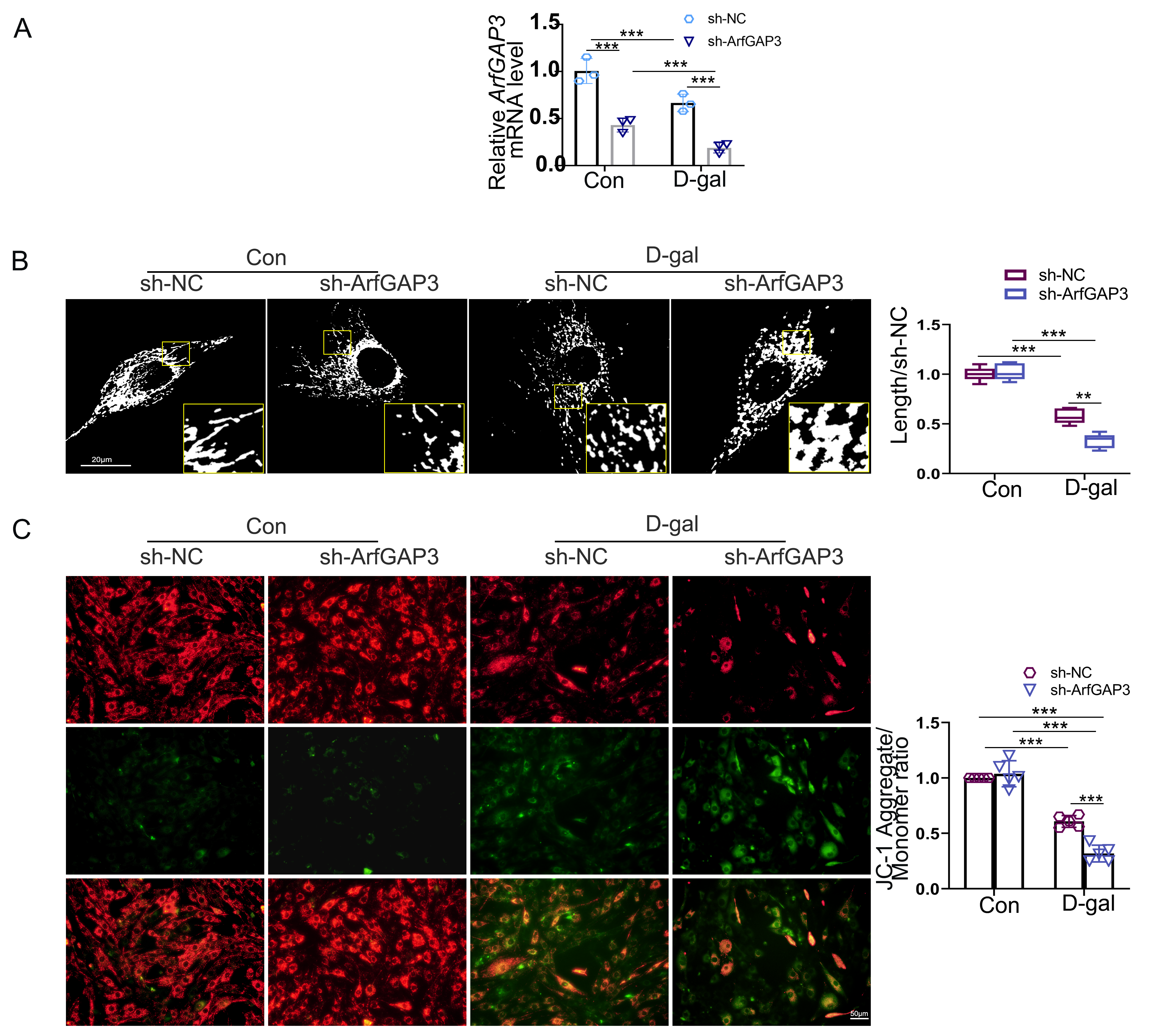

Supplement: Supplementary file 4 — Figure S4 (A) The mRNA level of ArfGAP3 in C2C12 myoblasts. (B) Representative immunofluorescence micrographs transferred to 8 bits images for quantification of C2C12 myoblasts. (C) Representative fluorescence images of MMP and quantification after incubation with JC‐1 in C2C12 myoblasts. Data were expressed as the mean ± SD and analysed using one‐ or two‐way ANOVA. **p < 0.01/ ***p < 0.001 vs. Con group. [file JCSM-16-e13725-s006.tif]

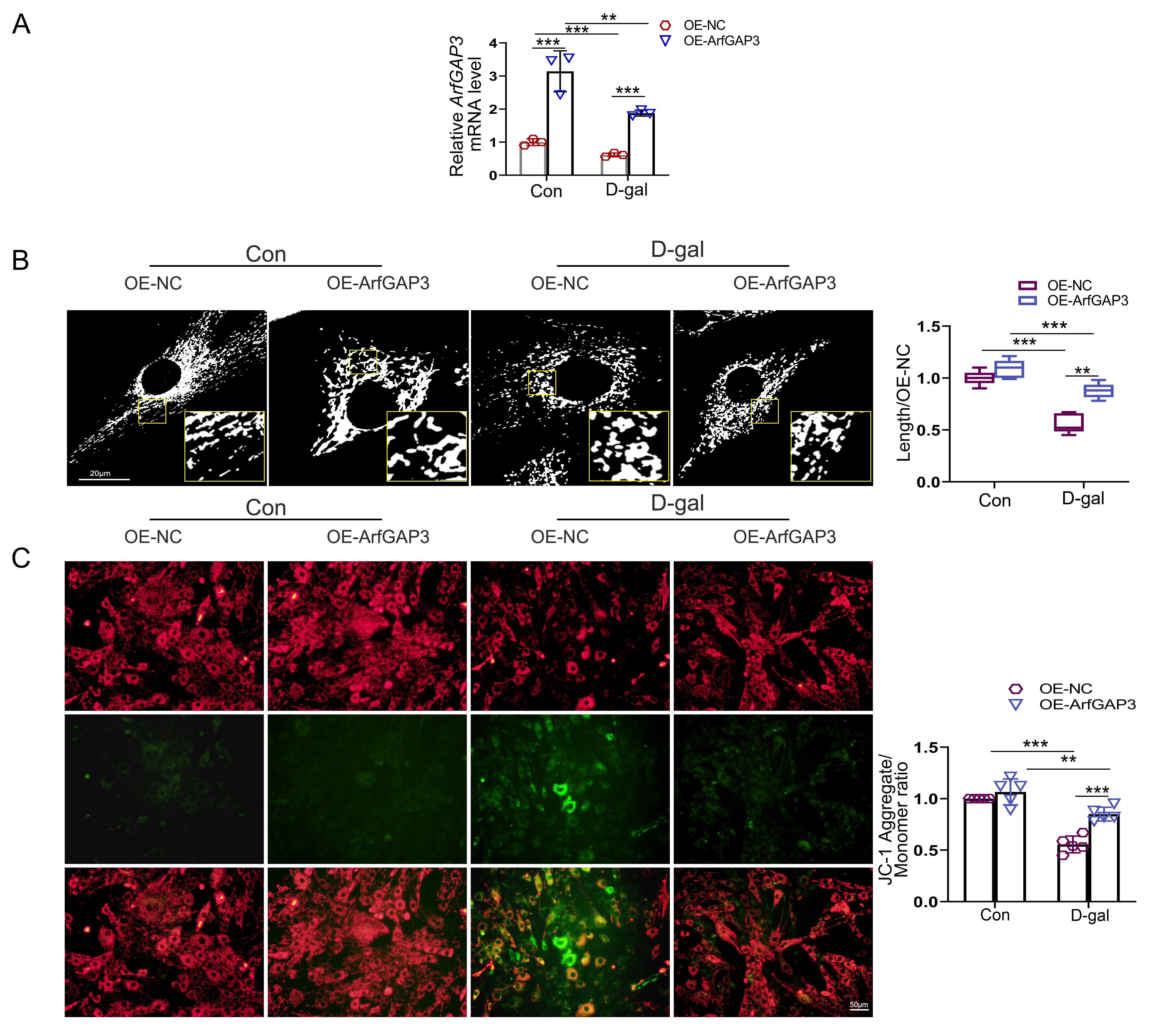

Supplement: Supplementary file 5 — Figure S5 (A) The mRNA levels of ArfGAP3 in C2C12 myoblasts after plasmid transfection with or without D‐gal treatment for 48 h. (B) Representative immunofluorescence micrographs transferred to 8 bits images for quantification of C2C12 myoblasts. (C) Representative fluorescence images of MMP and quantification after incubation with JC‐1 in C2C12 myoblasts. Data were expressed as the mean ± SD and analysed using one‐ or two‐way ANOVA. **p < 0.01/ ***p < 0.001 vs. Con group. [file JCSM-16-e13725-s005.tif]

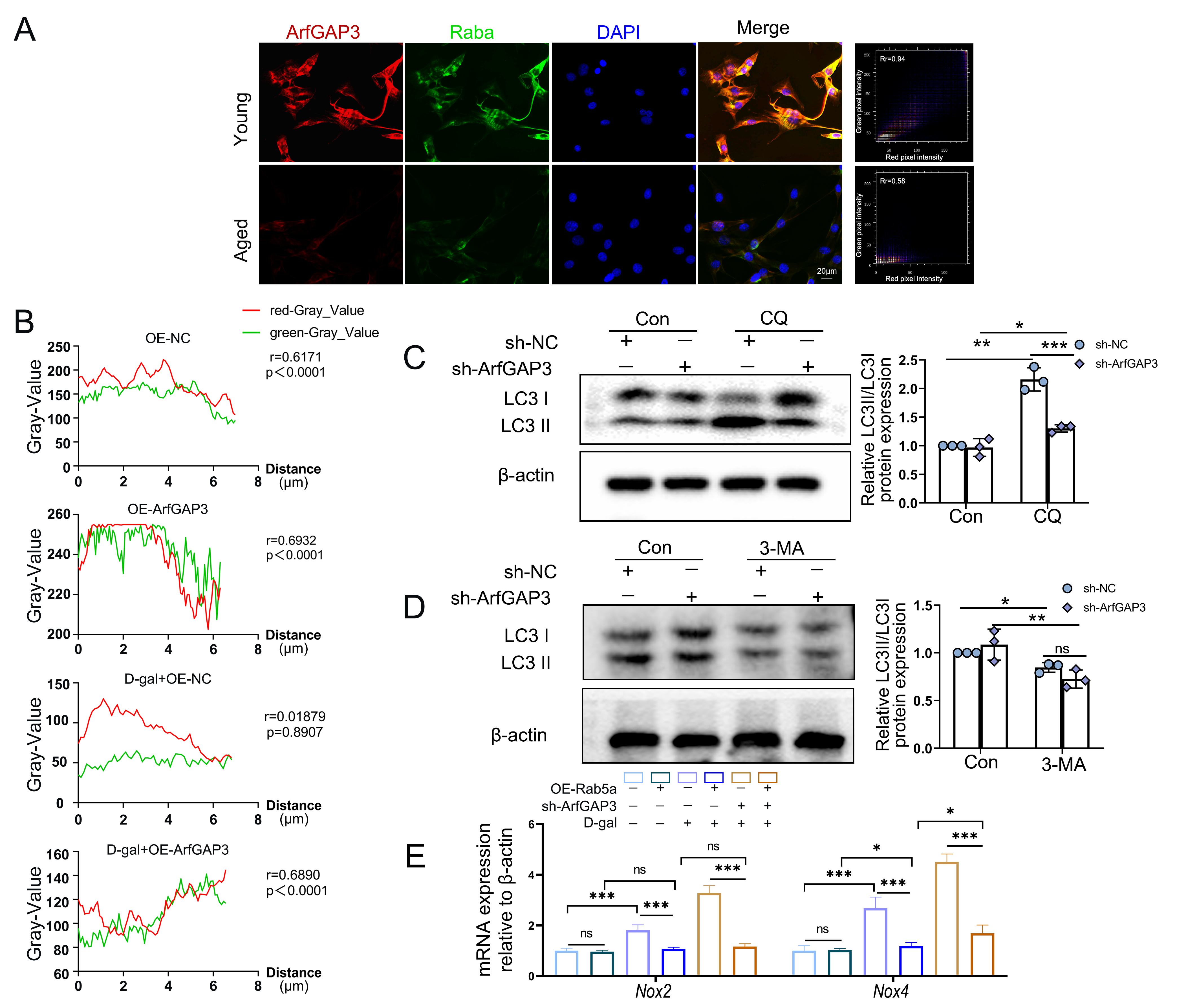

Supplement: Supplementary file 6 — Figure S6 (A) Images of immunofluorescence staining to present spatial expression patterns of ArfGAP3 (red) and Rab5a (green) in young and aged C2C12 cells. Scale bar = 20 μm. (B) The co‐localization curves that represented fluorescence intensity profiles were calculated from images. (C) Western blot analysis of LC3 proteins in C2C12 myoblasts treated with 10 mM chloroquine (CQ) and 2 mM 3‐methyladenine (3‐MA) (D) after ArfGAP3 knockdown. (E) The mRNA levels of Nox2 and Nox4. All data were presented as mean ± SD. All analyses were done using one‐ or two‐way ANOVA. *p < 0.05, **p < 0.01, ***p < 0.001. [file JCSM-16-e13725-s003.tif]

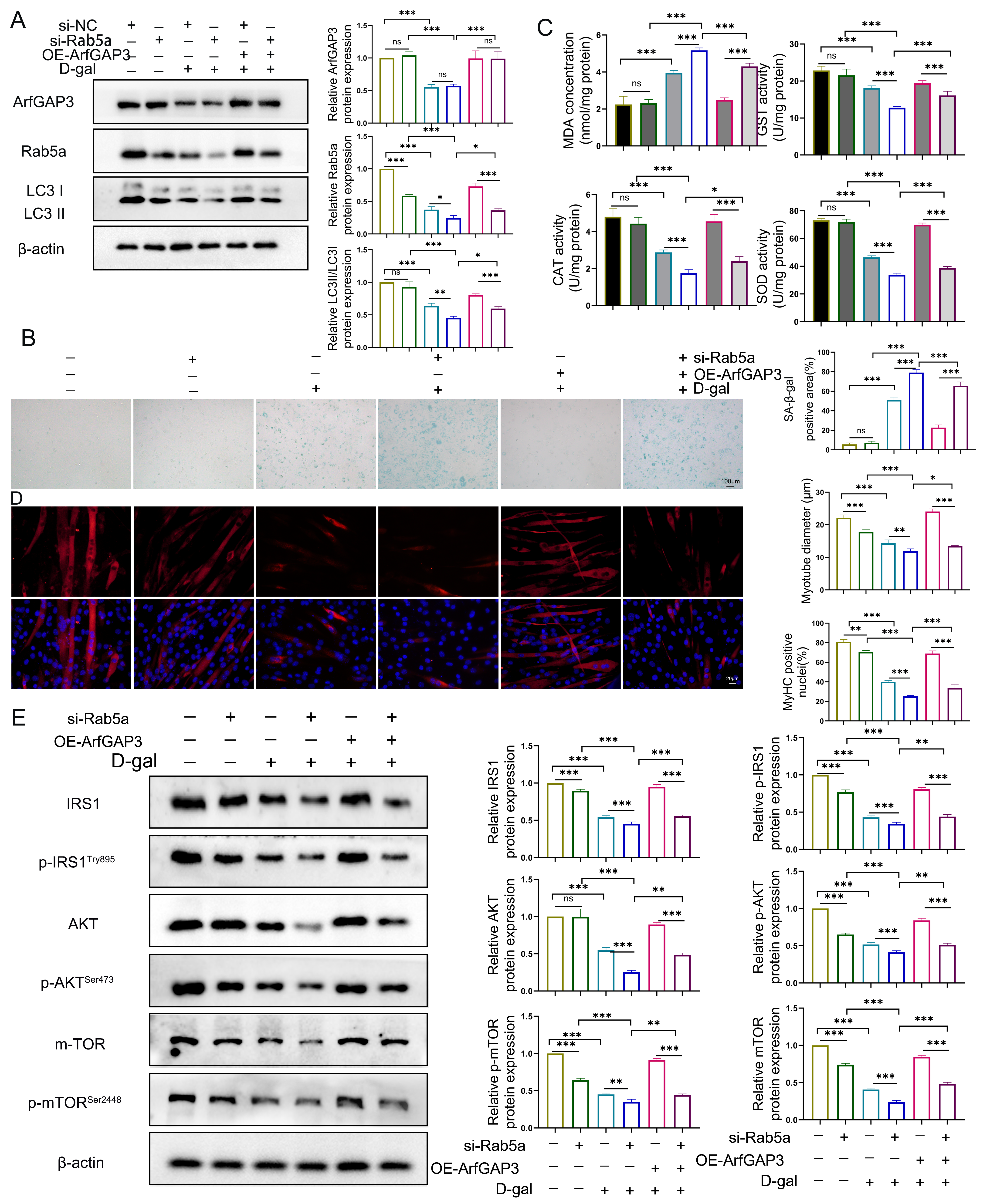

Supplement: Supplementary file 7 — Figure S7 (A) Western blot analysis and quantification of ArfGAP3, Rab5a and LC3 II/LC3I ratio levels. (B) SA‐β‐gal staining and quantification in C2C12 myoblasts with Rab5a knockdown in the presence of 20 g/L D‐gal; scale bar = 100 μm. (C) Contents of MDA in C2C12 cells and activities of antioxidant enzymes including CAT, GST, and SOD. (D) Immunofluorescent staining for MyHC in myotubes after differentiation for 5 days and quantification for myotube diameter and the percentage of MyhC‐positive nuclei (differentiation index). Scale bar = 20 μm. (F) Western blot analysis and respective quantification for MyhC, MyoG and MyoD after differentiation for 5 days. Data were presented as mean ± SD. Statistical analyses were conducted using one‐way ANOVA. *p < 0.05, **p < 0.01, ***p < 0.001. [file JCSM-16-e13725-s002.tif]

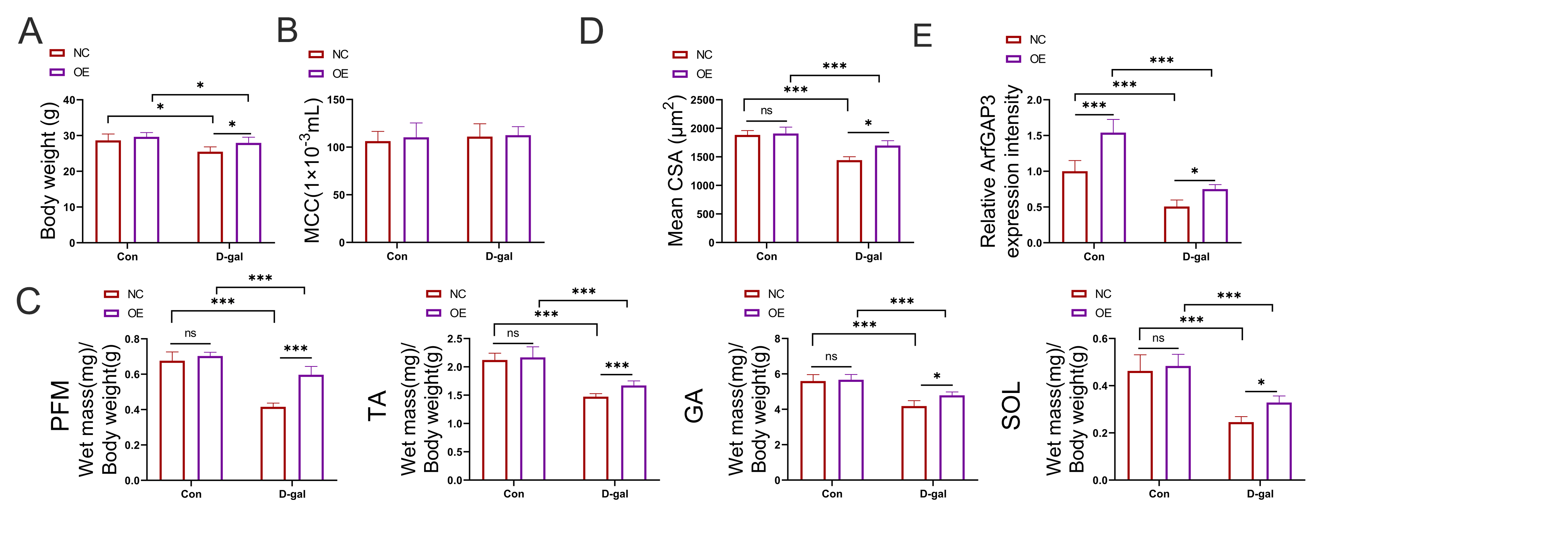

Supplement: Supplementary file 8 — Figure S8 (A) Body weight of mice in NC and OE group with or without D‐gal treatment for 4 months. (B) Average maximum cystometric capacity (MCC) of mice. (C) Wet weights of TA, GA, SOL and PFM muscles in NC and OE group with or without D‐gal treatment for 4 months. (D) The CSA of muscle fibres measured by ImageJ software. (E) The quantification for ArfGAP3 from IHC images in mouse PFM muscle (magnification 200×; scale bar = 50 μm; (bottom panels). [file JCSM-16-e13725-s001.tif]

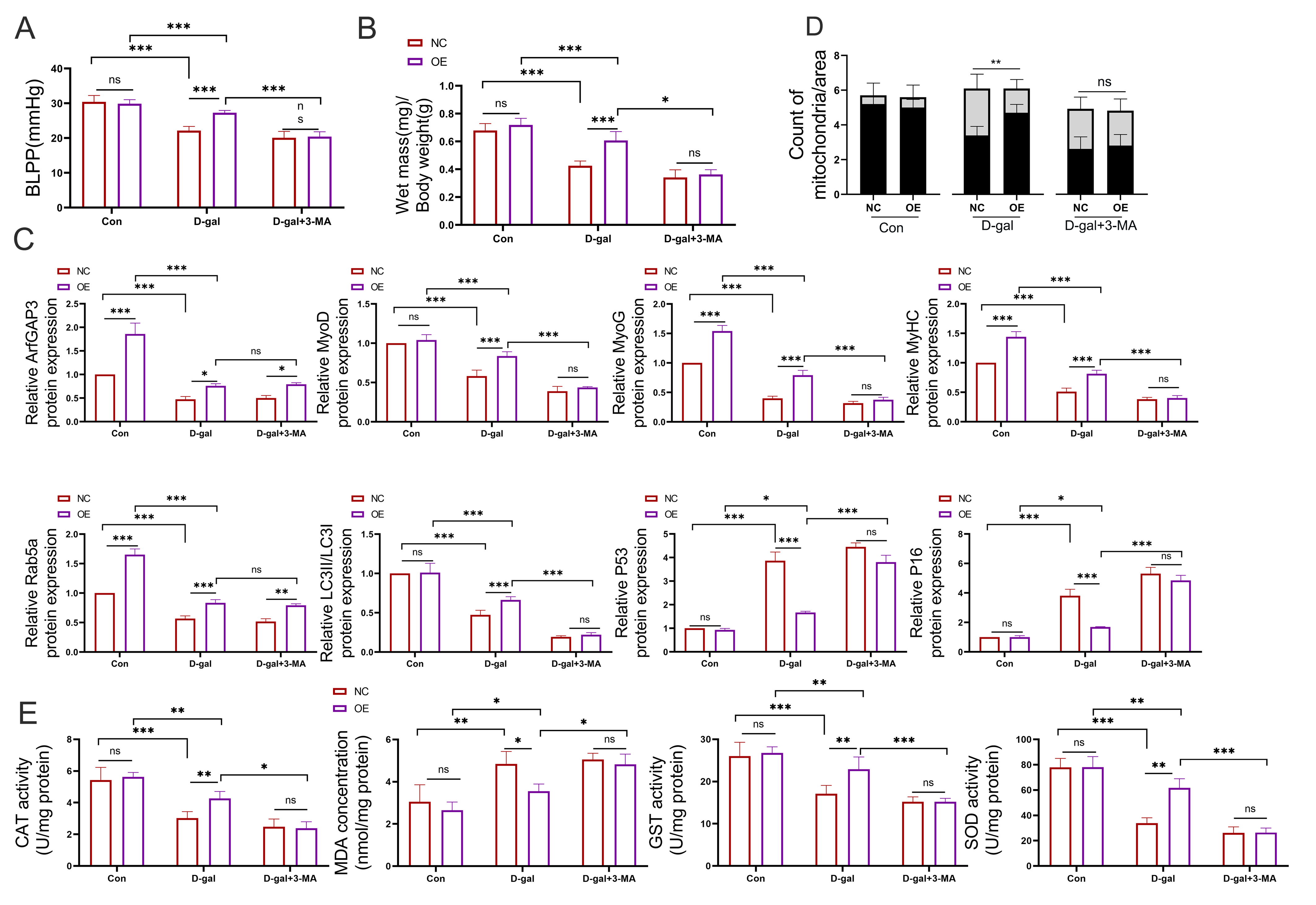

Supplement: Supplementary file 9 — Figure S9 (A) The average BLPP and MCC(B) values of mice. (C) Wet weights of PFM, TA, GA and SOL muscles. (PFM: pelvic floor muscle, GA: gastrocnemius, TA: tibialis anterior, and SOL: soleus) (F) Quantification of the count of damaged and normal mitochondria in PFM of TEM analysis. (G) Assessments for MDA content and the activity of antioxidant enzymes, including CAT, GST and SOD. All data were presented as mean ± SD. All analyses were done using one‐ or two‐way ANOVA. *p < 0.05, **p < 0.01, ***p < 0.001. [file JCSM-16-e13725-s004.tif]
