# Supplementary material for: ArfGAP3 Protects Mitochondrial Function and Promotes Autophagy Through Rab5a‐Mediated Signals in Ageing Skeletal Muscle
Source: J Cachexia Sarcopenia Muscle. 2025 Feb 17;16(1):e13725. doi: 10.1002/jcsm.13725 (PMC11832210; doi:10.1002/jcsm.13725)
Supplement: Supplementary file 10 — Table S1 Primer sequences for mouse genes. [file JCSM-16-e13725-s010.docx]

**Supplementary Materials**

**Materials and Methods**

**Bioinformatic analysis**

To examine expression levels of ArfGAP3 in skeletal muscle during aging process, we retrieved datasets related to “skeletal muscle aging” from the Gene Expression Omnibus (GEO) database (https://www.ncbi.nlm.nih.gov/geo/) and public dataset GSE117525 were selected and analyzed by R4.1.0 software. Briefly, skeletal muscles (*Musculus vastus lateralis*) were obtained from 53 young, 73 healthy older subjects, and 61 frail older subjects. Precise information about the grouping and processing methods were described in the GEO database.

**Cell culture and treatment**

C2C12 myoblasts were obtained from Cobioer Biosciences Co., Ltd. (Nanjing, China) and cultured in proliferation medium (PM) (Dulbecco’s modified Eagle’s medium (DMEM) + 10% fetal bovine serum (FBS)+ 1% penicillin‒streptomycin (P/S)) with 5% CO2 at 37 °C. For the myogenic differentiation process, the medium was changed to differentiation medium (DM) (DMEM + 2% horse serum + 1% P/S) for 5 days.

**Replicatively aged C2C12 model**

To investigate skeletal muscle cell aging, we employed a model of multiple population doublings of C2C12 myoblasts, which was previously described as a potential model to investigate muscle cell senescence (high passage 48–50)[S1,S5].Briefly, C2C12 cells were seeded at 1×10^6^ cells in T75 flasks in growth medium (DMEM supplemented with 10% FBS and 1% penicillin-streptomycin) until 70–80% confluent at 37°C in a humidified atmosphere with 5% CO2. The cells were then trypsinized and seeded onto new T75 flasks at a density of 1×10^6^ cells. The cycle was carried out 20 times over a 50-day period, yielding a batch of cells that had undergone multiple population doublings. We subsequently characterized the model in terms of the senescence-associated β-galactosidase (SA-β-gal) activity and senescence-associated markers P16 and P53.

**JC-1**

Following treatment, C2C12 cells were washed with PBS and incubated with JC-1 dye in culture medium at 37°C for 30 minutes. After incubation, cells were washed again with PBS and added 1mL culture medium. For the positive control group, C2C12 cells were treated with 10 µM CCCP (carbonyl cyanide m-chlorophenyl hydrazone, a mitochondrial uncoupler that rapidly depolarizes mitochondria) for 20 minutes with subsequent detection of mitochondrial membrane potential as described above. Images were acquired using a fluorescence microscope to visualize JC-1 aggregates (red) and monomers (green), representing mitochondrial polarization and depolarization.

**Cell proliferation**

After the indicated treatment, the proliferation rates of C2C12 myoblasts were measured using a BeyoClick™ EdU Cell Proliferation Kit with Alexa Fluor 594 (Beyotime Biotechnology, China, C0078S) following the manufacturer's protocol. Briefly, myoblasts were incubated with 1 mL of PM containing 20 μM EdU (final concentration 10 μM) for 4 h and fixed with 4% paraformaldehyde for 15 min. The myoblasts were washed twice with 3% BSA, incubated with 0.5% Triton X-100, washed twice and incubated with Click Additive Solution for 30 min at room temperature. After incubation with DAPI for an additional 10 min in the dark, the myoblasts were imaged at 200× magnification under a microscope (Olympus, Japan). Six fields for each sample were randomly captured. EdU-positive cells were counted using ImageJ software.

**Gene Expression Analyses**

Total RNA was isolated from PFM or C2C12 myoblasts using Invitrogen^TM^ TRIzol total RNA extraction reagent (ThermoFisher Scientific) according to the manufacturer’s instructions. Reverse transcription was performed using a Hifair® II 1st Strand cDNA Synthesis Kit (YEASEN, China) for RT‒PCR according to the manufacturer’s instructions, starting with 1 μg of total RNA. Primers (Sangon Biotech, China) were added, and the primer sequences are listed in Table S1. Quantitative real-time PCR was performed with the Hieff® qPCR SYBR Green Master Mix (Low Rox Plus) Kit (YEASEN, China) using the CFX96 Trademark Real-time PCR detection system (Bio-Rad, California, USA). Melting curves for each primer pair were assessed to ensure specific amplification. The transcript levels were normalized to those of β-actin. The data were analyzed by the 2-ΔΔCt threshold cycle method.

**Co-immunoprecipitation**

Co-Immunoprecipitation (Co-IP) assay was performed in C2C12 myoblasts using Dynabeads™ Co-Immunoprecipitation Kit (ThermoFisher Scientific, USA). According to the manufacturer’s manuals, the protein extracted with IP Lysis Buffer was subject to beads premixed with antibodies of ArfGAP3 or IgG. The immunoprecipitated protein complex was separated from beads after several washes, followed by the identification for partners of ArfGAP3 by immunoblots.

**Western blotting**

Proteins were extracted from PFM and C2C12 myoblasts with ice-cold RIPA buffer (Beyotime, China), separated on 10% SDS‐polyacrylamide gels, and transferred onto PVDF membranes (Millipore, USA), which were soaked in 5% skim milk (overnight, 4 °C), washed, and then exposed overnight to primary antibodies. Subsequently, the membranes were washed and incubated with the corresponding goat anti-mouse (Proteintech, China) or anti-rabbit (Proteintech, China) secondary antibodies at room temperature for 1 h. After being washed with TBST, enhanced chemiluminescence reagent (Millipore, USA) was added, and the images were acquired and quantified using ImageJ software. The list of primary antibodies utilized is reported in the Supplementary Table 2.

**Muscle injury and regeneration**

At the respective experimental endpoints delineated above, mice were anesthetized throughout modeling using isoflurane prior to the injection procedure. Subsequently, 50 µl of cardiotoxin (CTX) solution (10 μM; Sigma-Aldrich) was injected into the left PFM, while the right PFM received injections of physiological saline. Following a 3-day interval, the mice were sacrificed to collect tissue samples for subsequent analysis.

**Evans Blue Dye Assays**

Mice were intraperitoneally injected with Evans blue dye (10 mg/mL) at a dosage of 5 μL/g of body weight 24 hours before sacrifice, as described previously [S6]. For Evans Blue dye fluorescence analysis, cryosections (8-μm thick transverse sections of frozen muscle cut in a cryostat at −20 °C) were mounted on coverslips with buffered glycerol and examined using fluorescent microscopy.

**Immunohistochemical staining (IHC)**

For IHC analyses, deparaffinized and hydrated sections were incubated in citric acid buffer at 95 °C for 20-30 min. After incubation with 3% hydrogen peroxide for 20 min and incubation with 10% goat serum for 30 min at 37 °C, the sections were treated with primary antibodies against ArfGAP3 (1:100, Proteintech, 15293-1-AP) overnight at 4 °C. After washing with PBS, the sections were incubated with secondary antibodies for 30 min at 37 °C. After adding diaminobenzidine solution (Wuhan Servicebio Technology G1212, China), the reactions were observed under a microscope and stopped in distilled water when the target area turned yellow. Finally, the nuclei were stained with hematoxylin, and the slides were dehydrated and mounted. The images were acquired with an Olympus BX53 digital imaging system at 200× magnification (Olympus Corporation, Tokyo, Japan) and analyzed with ImageJ software.

**Transmission electron microscopy**

Within 1 minute after the mice were sacrificed, the isolated PFM were fixed in 2.5% glutaraldehyde buffered fixative precooled at 4 °C and randomly cut into five to six tissue blocks (1 mm^3^) in the same area between groups. All samples were subsequently processed for transmission electron microscopy (TEM, HITACHI HT7700, Tokyo, Japan) at the Ultrapathological Center of Renmin Hospital of Wuhan University. From each section, ten fields of images were randomly captured at a magnification of 12.0K×.

**Immunofluorescence staining**

C2C12 myoblasts cultured in 6-well plates were fixed with 4% paraformaldehyde for 15 min after the indicated treatment. Then, the myoblasts were permeabilized with 0.2% Triton X‐100 for 10 min and blocked with 5% BSA for 1 h. Next, the myoblasts were incubated with primary antibody solution overnight at 4 °C. A fluorescent secondary antibody was added and incubated for 1 h at room temperature, and the nuclei were stained with DAPI for 5 min. The myoblasts were washed with PBS three times between each step. For colocalization of ArfGAP3 and Rab5a, images were acquired by confocal laser scanning microscopy (Olympus FV1200). ImageJ software was used to measure the fluorescence intensity and the average diameters of myotubes for the captured images.

**Detection of antioxidant capacity**

The concentration of malondialdehyde (MDA) was measured with a *Lipid Peroxidation MDA Assay Kit* (Beyotime Biotechnology, Nanjing, China) according to the manufacturer’s protocol. The activity of superoxide dismutase (SOD) and catalase (CAT) was measured with a *Total Superoxide Dismutase Assay Kit with WST-8* and a *Catalase Assay Kit* (Beyotime Biotechnology, Nanjing, China). A *Glutathione S-transferase (GST) Activity Assay Kit* (Solarbio, Beijing, China) was used for GST activity detection. Briefly, cell lysates or muscle homogenates were prepared according to the instructions of the corresponding kit, the protein concentrations were measured using a BCA protein assay kit (Beyotime), and then the absorbance or fluorescence was measured with a multifunctional microplate reader (PerkinElmer, America) at the corresponding wavelength.

**Autophagy analyses**

Autophagic flux was assessed with mRFP-GFP-LC3 adenoviral particles (HanBio, Shanghai, China) as described. C2C12 myoblasts were infected with adenoviral particles, and images were acquired with a confocal laser scanning microscope (Olympus FV1200). ImageJ software was used to measure the fluorescence intensity. Puncta structures with GFP–RFP and/or RFP signals were quantified in more than 80 myoblasts per group, and the degree of autophagosome maturation was expressed as the percentage of puncta with red color.

**Table S1. Primer sequences for mouse genes**

| Gene | Forward Primer (5’→3’) | Reverse Primer (5’→3’) |
| --- | --- | --- |
| Arfgap1 | CGGGCACAGGATGAGAATAATG | CTGACCCACTGAGGATTGAAC |
| Arfgap2 | CCGAGCAAGACCGAAATCCAG | GCGCCACAATCGAAACAGG |
| Arfgap3 | TGGGGTGTTTCTCTGCATTGA | GGAACCACGACCAGTTTGAAT |
| Rab5a | GCTAATCGAGGAGCAACAAGAC | CCAGGCTTGATTTGCCAACAG |
| Nox2 | AGTGCGTGTTGCTCGACAA | GCGGTGTGCAGTGCTATCAT |
| Nox4 | TGCCTGCTCATTTGGCTGT | CCGGCACATAGGTAAAAGGATG |
| Rab1a | TTACTTCTGATTGGCGATTCTGG | TGGGCTCCTCTGTAATAACTGG |
| Rab7a | AGGCTTGGTGCTACAGGAAAA | CTTGGCCCGGTCATTCTTGT |
| Rab11a | TGGGAAAACAATAAAGGCACAGA | ATGTGAGATGCTTAGCAATGTCA |
| Rab34 | GGGAGACCTATCTGTGGGGAA | TGTCCCAAAGTTGGAGACTGA |
| β-actin | ATGCTCCCCGGGCTGTAT | CATAGGAGTCCTTCTGACCCATTC |

**Figure S1.**


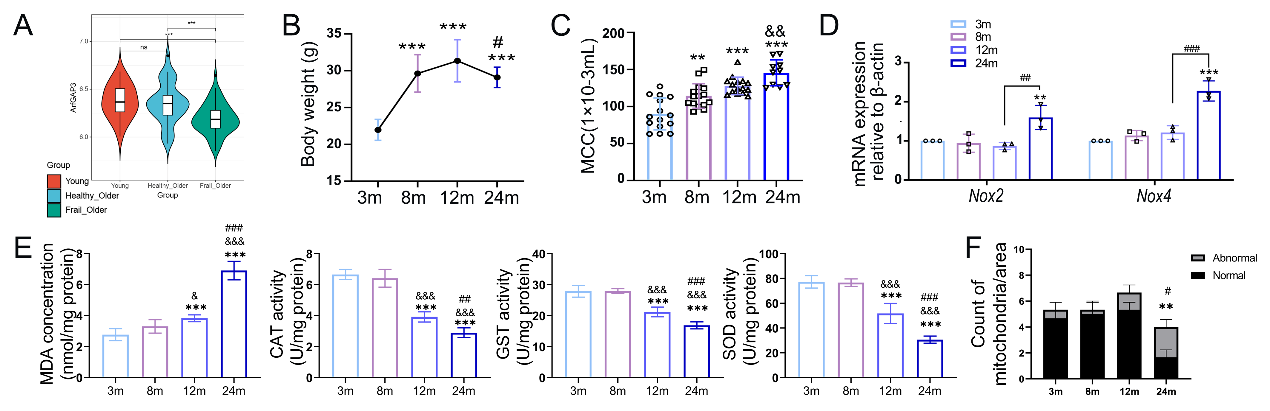


Figure S1.

(A) Comparison analysis of ArfGAP3 expression in 53 young, 73 healthy older and 61 frail older subjects in GSE117525. (B) The curves for body weight of 3-, 8-, 12- and 24-month-old mice (n=15). (C) Average maximum cystometric capacity (MCC) of mice in the four groups (n=15). (D) The mRNA levels of the oxidative stress-related genes (*Nox2* and *Nox4*) by qRT-PCR. (E) Assessments of MDA contents in C2C12 cells and activities of antioxidant enzymes including CAT, GST, and SOD. (F) Quantification for the count of damaged and normal mitochondria in PFM from TEM analysis. All data were presented as mean ± SD (n=3). Data were analyzed using one-way ANOVA. *p < 0.05 / **p < 0.01 / ***p < 0.001 vs. 3 mon group; &< 0.05 /&& < 0.01 / &&&p < 0.001 vs.8 mon group; #p < 0.05 / ## < 0.01 / ### p < 0.05 vs. 12 mon group.

**Figure S2.**

**
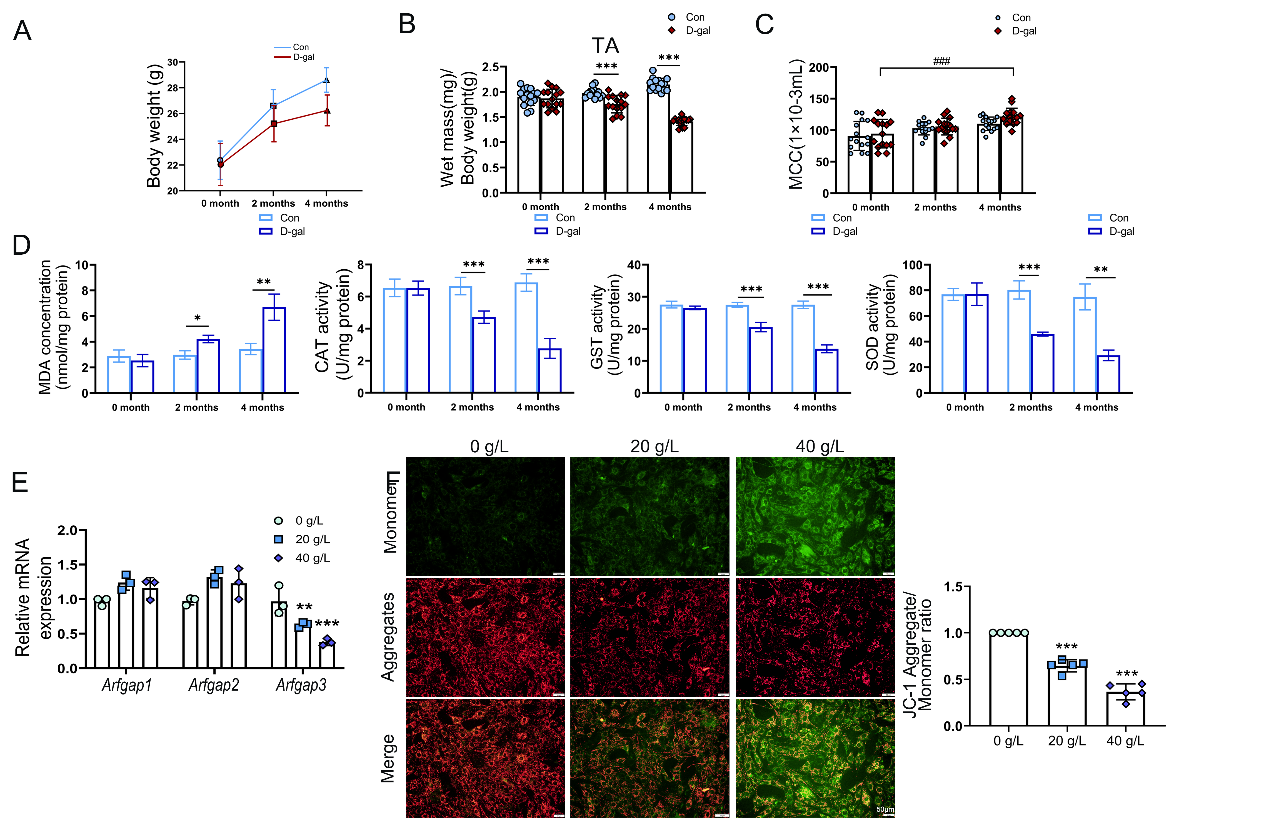
**

Figure S2.

(A) Body weight curves from control (Con) and D-gal-treated (D-gal) mice (n =15). (B) Weights assessment of Anterior (TA) muscle (n=15). (C) Average maximum cystometric capacity (MCC) of mice (n=15). (D) Assessments of MDA contents in C2C12 cells and activities of antioxidant enzymes including CAT, GST, and SOD. (E) qRT-PCR analysis of the expression of *Arfgap1, Arfgap2* and *Arfgap3*. *β-actin* was used as the loading control (n=3). (F) Representative fluorescence images of MMP and quantification after incubation with JC-1 in C2C12 myoblasts. Red fluorescence represents JC-1 aggregates in healthy mitochondria, whereas green fluorescence represents JC-1 monomers, indicating MMP dissipation (n=5). Merged images represent colocalization of the JC-1 aggregates and JC-1 monomers (scale bar = 50 μm). Data were expressed as the mean ± SD, and analyzed using one-way ANOVA. **p < 0.05 / **p < 0.01 / ***p < 0.001 vs. Con group or 0 g/L group; &&p < 0.01 / &&&p < 0.001 vs. 2 months group.

**Figure S3**


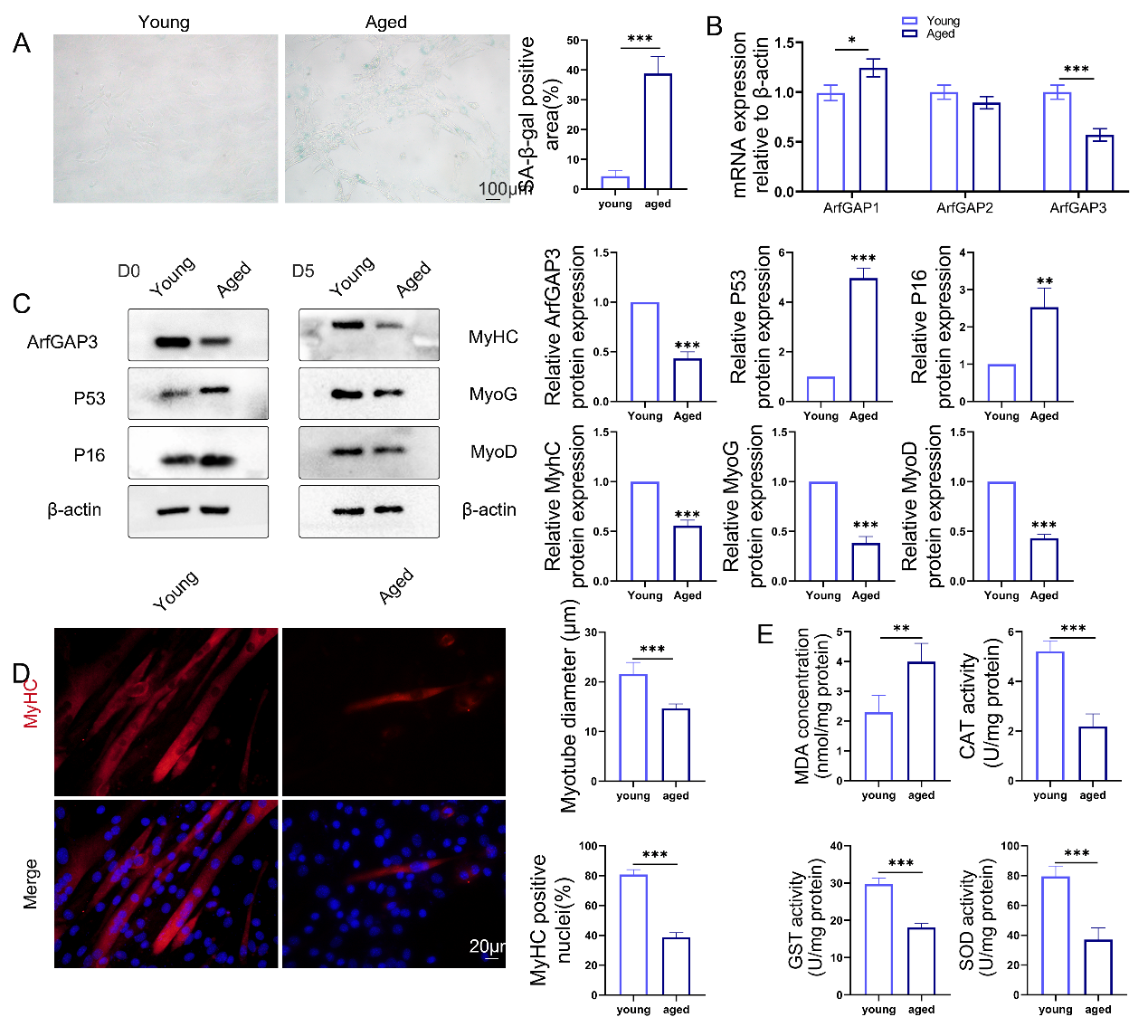


Figure S3.

(A) SA-β-gal staining and quantification for young and aged C2C12 cells of a model of multiple population doublings. Scale bar = 100 μm. (B) qRT-PCR analysis of the expression of Arfgap1, Arfgap2 and Arfgap3. β-actin was used as the loading control. (C) Western blot analysis and quantification of ArfGAP3, P16 and P53 proteins in young and aged C2C12 cells (D0) and protein levels of MyHC, MyoG and MyoD after differentiation for 5 days (D5). (D) Immunofluorescent staining for MyHC in young and aged C2C12 cells after differentiation for 5 days and quantification for myotube diameter and the percentage of MyhC-positive nuclei (differentiation index). Scale bar = 20 μm. (E) Assessments of MDA contents in C2C12 cells and activities of antioxidant enzymes including CAT, GST, and SOD. Data were expressed as the mean ± SD, and an unpaired two-tailed Student’s t test was used to analyze the statistical significance between two groups. ∗p < 0.05, ∗∗p < 0.01, ∗∗∗p < 0.001.

**Figure S4**

**
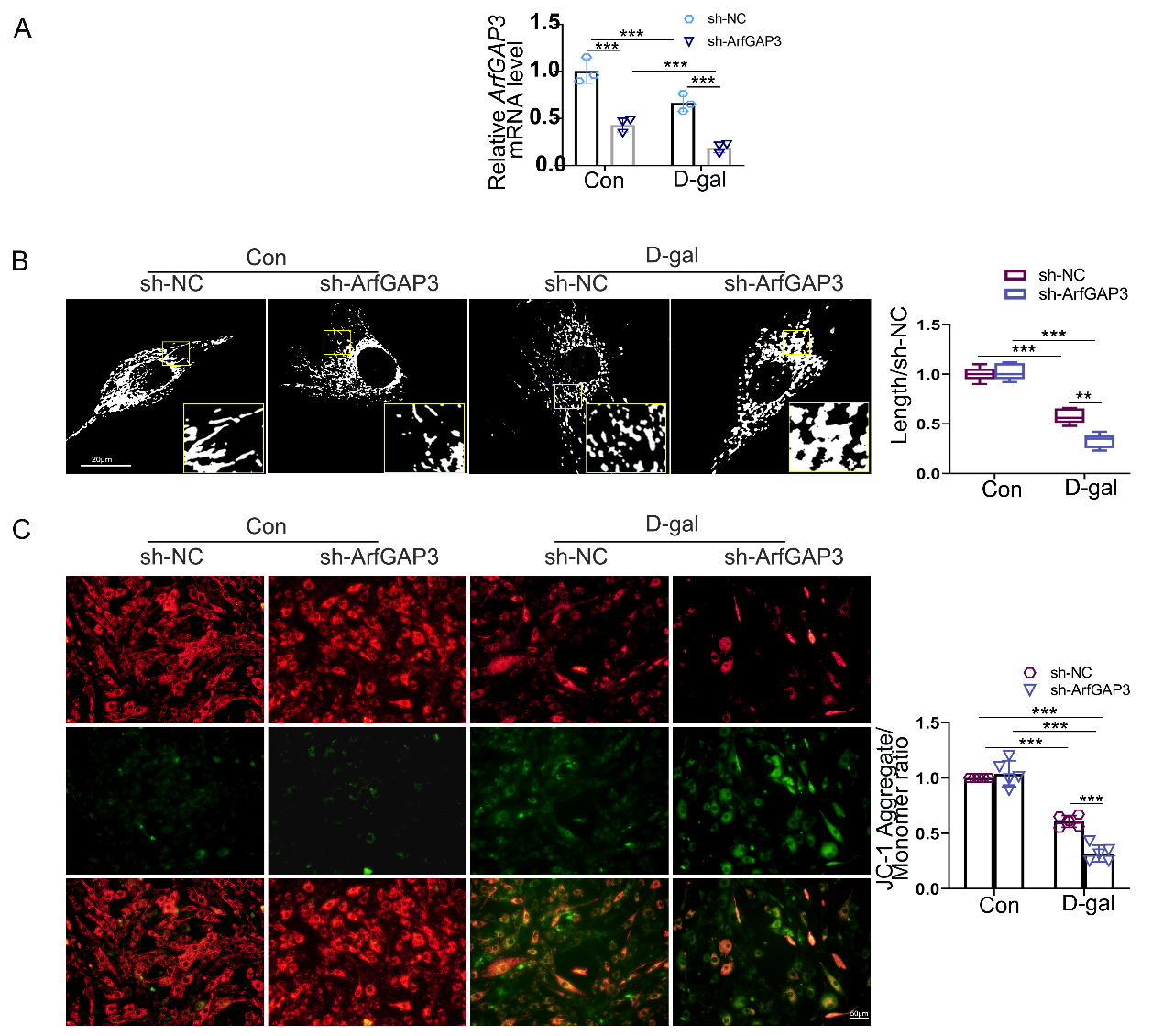
**

Figure S4

(A) The mRNA level of ArfGAP3 in C2C12 myoblasts. (B) Representative immunofluorescence micrographs transferred to 8 bits images for quantification of C2C12 myoblasts. (C) Representative fluorescence images of MMP and quantification after incubation with JC-1 in C2C12 myoblasts. Data were expressed as the mean ± SD and analyzed using one- or two-way ANOVA. **p < 0.01 / ***p < 0.001 vs. Con group.

**Figure S5**


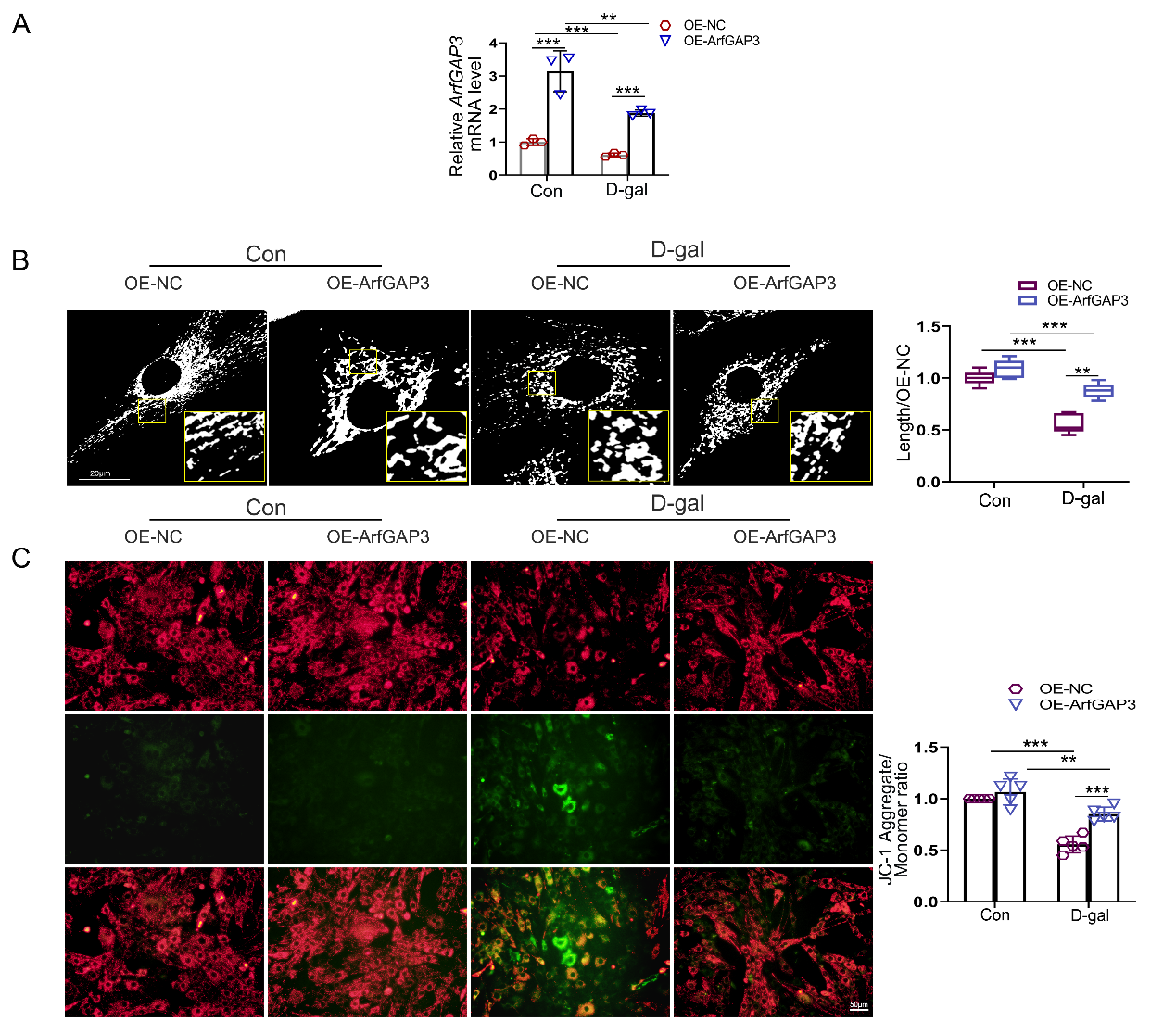


Figure S5

(A) The mRNA levels of ArfGAP3 in C2C12 myoblasts after plasmid transfection with or without D-gal treatment for 48 h. (B) Representative immunofluorescence micrographs transferred to 8 bits images for quantification of C2C12 myoblasts. (C) Representative fluorescence images of MMP and quantification after incubation with JC-1 in C2C12 myoblasts. Data were expressed as the mean ± SD and analyzed using one- or two-way ANOVA. **p < 0.01 / ***p < 0.001 vs. Con group.

**Figure S6**


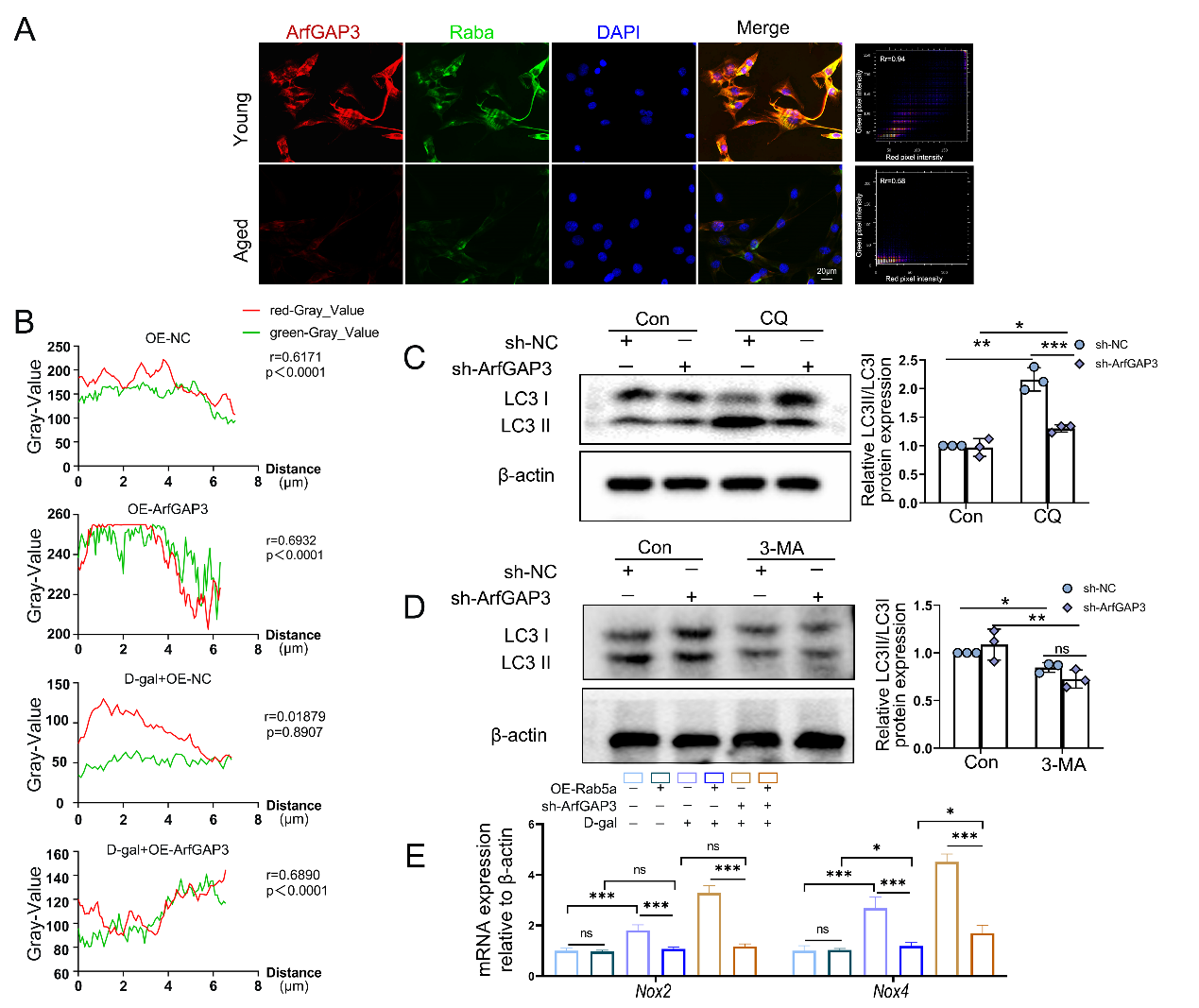


Figure S6 (A) Images of immunofluorescence staining to present spatial expression patterns of ArfGAP3 (red) and Rab5a (green) in young and aged C2C12 cells. scale bar = 20 μm. (B) The co-localization curves that represented fluorescence intensity profiles were calculated from images. (C) Western blot analysis of LC3 proteins in C2C12 myoblasts treated with 10mM chloroquine (CQ) and 2mM 3-methyladenine (3-MA) (D) after ArfGAP3 knockdown. (E) The mRNA levels of *Nox2* and *Nox4*. All data were presented as mean ± SD. All analyses were done using one- or two-way ANOVA. *p < 0.05, **p < 0.01, ***p < 0.001.

**Figure S7**


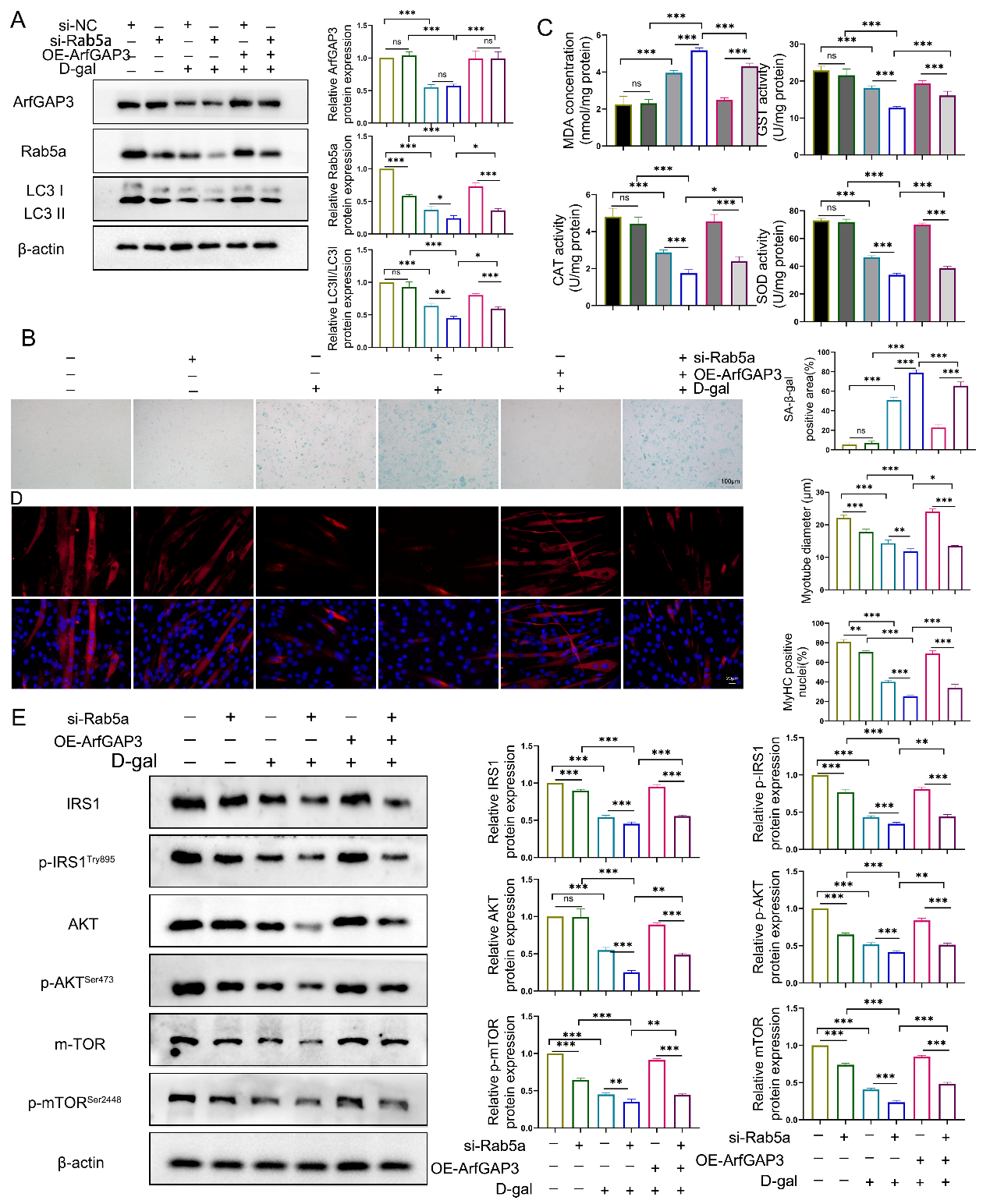


Figure S7

1. Western blot analysis and quantification of ArfGAP3, Rab5a and LC3 II/LC3I ratio levels. (B) SA-β-gal staining and quantification in C2C12 myoblasts with Rab5a knockdown in the presence of 20g/L D-gal; scale bar = 100 μm. (C) Contents of MDA in C2C12 cells and activities of antioxidant enzymes including CAT, GST, and SOD. (D) Immunofluorescent staining for MyHC in myotubes after differentiation for 5 days and quantification for myotube diameter and the percentage of MyhC-positive nuclei (differentiation index). Scale bar = 20 μm. (F) Western blot analysis and respective quantification for MyhC, MyoG and MyoD after differentiation for 5 days. Data were presented as mean ± SD. Statistical analyses were conducted using one-way ANOVA. *p < 0.05, **p < 0.01, ***p < 0.001.

**Figure S8**


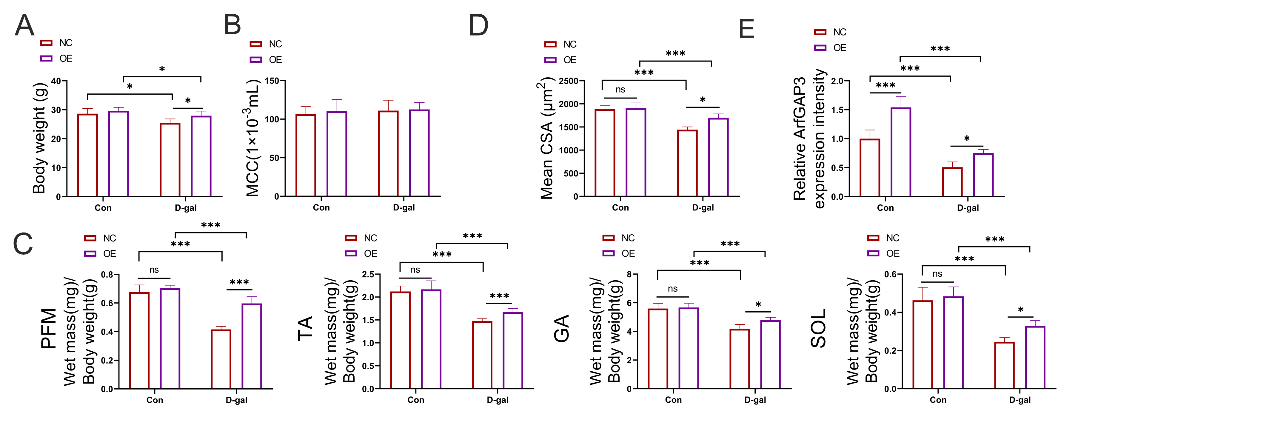


Figure S8 (A) Body weight of mice in NC and OE group with or without D-gal treatment for 4 months. (B) Average maximum cystometric capacity (MCC) of mice. (C) Wet weights of TA, GA, SOL and PFM muscles in NC and OE group with or without D-gal treatment for 4 months. (D) The CSA of muscle fibers measured by ImageJ software. (E) The quantification for ArfGAP3 from IHC images in mouse PFM muscle (magnification 200×; scale bar = 50 μm; (bottom panels).

**Figure S9**


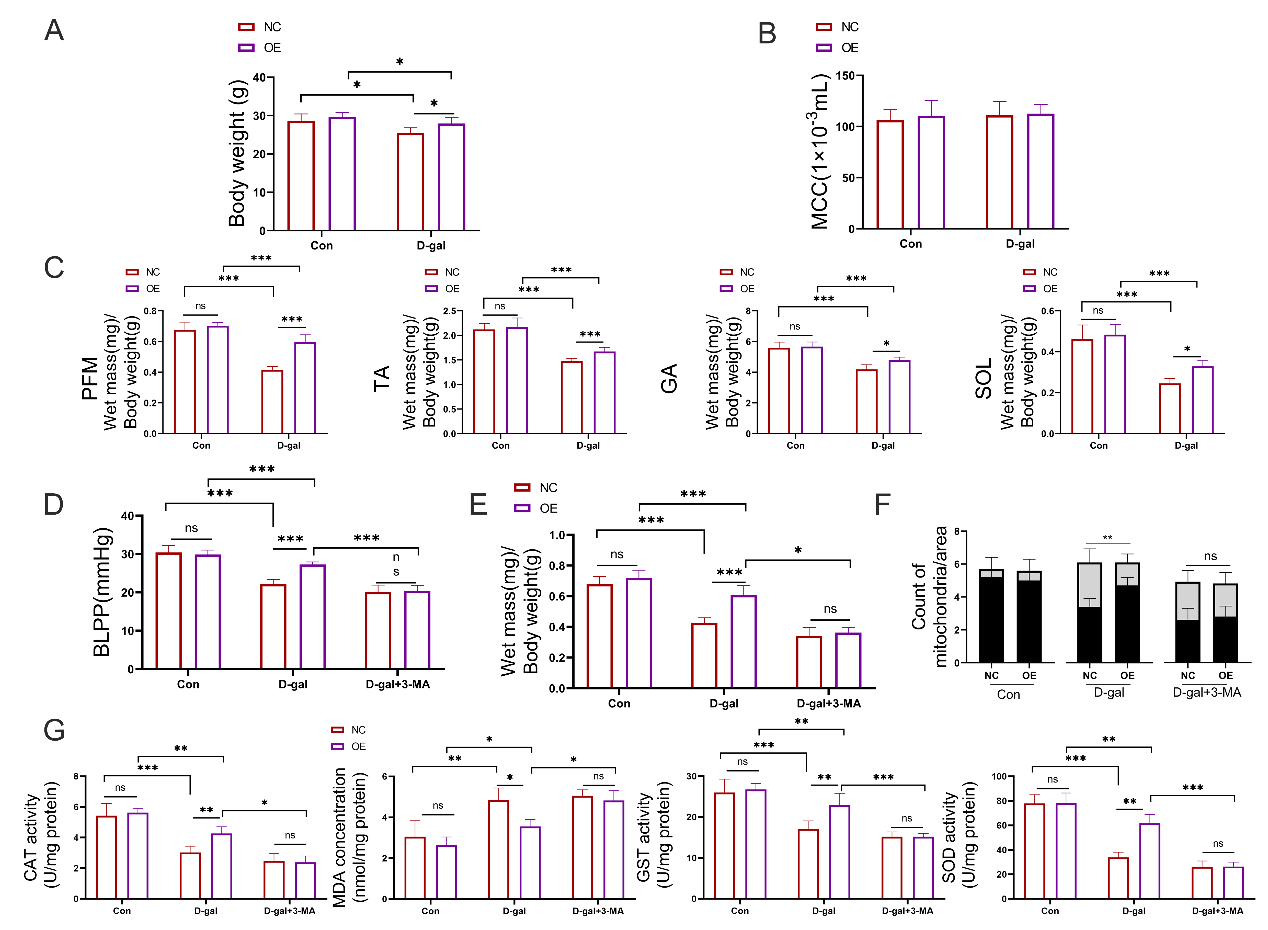


(A) The average BLPP and MCC(B) values of mice. (C) Wet weights of PFM, TA, GA and SOL muscles. (PFM: pelvic floor muscle, GA: gastrocnemius, TA: tibialis anterior, and SOL: soleus) (F) Quantification of the count of damaged and normal mitochondria in PFM of TEM analysis. (G) Assessments for MDA content and the activity of antioxidant enzymes, including CAT, GST and SOD. All data were presented as mean ± SD. All analyses were done using one- or two-way ANOVA. *p < 0.05, **p < 0.01, ***p < 0.001.

**References**

[1] Brown A D, Stewart C E, Burniston J G. Degradation of ribosomal and chaperone proteins is attenuated during the differentiation of replicatively aged C2C12 myoblasts[J]. J Cachexia Sarcopenia Muscle. 2022, 13(5): 2562-2575.

[2] Moustogiannis A, Philippou A, Taso O, et al. The Effects of Muscle Cell Aging on Myogenesis[J]. Int J Mol Sci. 2021, 22(7).
